# Maximizing response to intratumoral immunotherapy in mice by tuning local retention

**DOI:** 10.1038/s41467-021-27390-6

**Published:** 2022-01-10

**Authors:** Noor Momin, Joseph R. Palmeri, Emi A. Lutz, Noor Jailkhani, Howard Mak, Anthony Tabet, Magnolia M. Chinn, Byong H. Kang, Virginia Spanoudaki, Richard O. Hynes, K. Dane Wittrup

**Affiliations:** 1 Koch Institute for Integrative Cancer Research, Massachusetts Institute of Technology, Cambridge, MA 02142 USA; 2 Department of Biological Engineering, Massachusetts Institute of Technology, Cambridge, MA 02142 USA; 3 Department of Chemical Engineering, Massachusetts Institute of Technology, Cambridge, MA 02142 USA; 4 Research Laboratory of Electronics, Massachusetts Institute of Technology, Cambridge, MA 02142 USA; 5 McGovern Institute for Brain Research, Massachusetts Institute of Technology, Cambridge, MA 02142 USA; 6 Department of Biology, Massachusetts Institute of Technology, Cambridge, MA 02142 USA

**Keywords:** Cancer immunotherapy, Drug development, Computational models, Pharmacokinetics, Biomedical engineering

## Abstract

Direct injection of therapies into tumors has emerged as an administration route capable of achieving high local drug exposure and strong anti-tumor response. A diverse array of immune agonists ranging in size and target are under development as local immunotherapies. However, due to the relatively recent adoption of intratumoral administration, the pharmacokinetics of locally-injected biologics remains poorly defined, limiting rational design of tumor-localized immunotherapies. Here we define a pharmacokinetic framework for biologics injected intratumorally that can predict tumor exposure and effectiveness. We find empirically and computationally that extending the tumor exposure of locally-injected interleukin-2 by increasing molecular size and/or improving matrix-targeting affinity improves therapeutic efficacy in mice. By tracking the distribution of intratumorally-injected proteins using positron emission tomography, we observe size-dependent enhancement in tumor exposure occurs by slowing the rate of diffusive escape from the tumor and by increasing partitioning to an apparent viscous region of the tumor. In elucidating how molecular weight and matrix binding interplay to determine tumor exposure, our model can aid in the design of intratumoral therapies to exert maximal therapeutic effect.

## Abstract (web summary)

Understanding the pharmacokinetics of locally-injected drugs could aid in the design of immunotherapies to maximize their therapeutic effect. Here, by evaluating different IL-2 fusion proteins, the authors show that molecular weight and matrix binding affect anti-tumor immune response and report a pharmacokinetic framework to predict response to intratumoral IL-2 therapy.

## Introduction

Intratumoral administration enables the use of potent immune modulators to treat cancer. As a result, the number of trials investigating local administration of cancer therapies has boomed^1^. Modalities undergoing intratumoral testing^2,3^ include small molecules^4^, nucleic acids^5^, proteins^6^, viral-vector-based agents^7^, and cell therapies^8,9^.

Response to most local therapies hinges on tumor exposure^10^, which depends on a drug’s pharmacokinetic properties. Amongst the features that influence pharmacokinetics, molecular size governs several transport phenomena relevant to tumor retention (e.g. extravasation, diffusion, accessible interstitial volume)^11^. Moreover, molecular size varies tremendously across therapeutic modalities. Previous characterizations of the biodistribution of large locally injected particles has provided a pharmacokinetic perspective on nanoparticle (~10–100 nm), liposomal (~20 nm−1 µm), viral-vector (~100 nm), and cell-based therapies (~10–20 µm)^12,13^. However, for smaller-sized protein therapies (e.g. peptides, nanobodies, cytokines, antibodies) ranging from 1 to 10 nm in molecular radius, it remains unclear quantitatively how their differences in size impacts tumor exposure. For proteins, tumor retention is also affected by target binding. Drug pharmacokinetics seems intuitive, yet analyses outlining exactly how target binding and molecular size impacts the tumor exposure of intravenously injected biologics have proven pertinent to their design, optimization and clinical translation^14–21^. Intratumorally administered biologics would benefit from similar foundational pharmacokinetic analysis, which presently does not exist.

Pharmacokinetic insight can inform improvements to immune modulators, such as the cytokine interleukin-2 (IL-2). IL-2 is capable of eliciting durable tumor regression via natural killer (NK) and T cell activation but is plagued by severe systemic toxicity. Thus IL-2 has been clinically evaluated as a local therapy in different formats (e.g. wild-type^22,23^, PEGylated^24^, fused to fibronectin targeting diabody^25,26^, fused to GD2 binding antibody^27^, and fused to epithelial cell adhesion molecule binding antibody^28^). Using IL-2 as an example, here we report the role of its pharmacokinetics in controlling therapeutic efficacy after intratumoral injection and define strategies to maximize anti-tumor effect by tuning local retention. To tune local retention, we fuse IL-2 to proteins of different size and matrix-binding affinity. By varying these two attributes, we systematically alter the local pharmacokinetic features of IL-2 fusion proteins and define how each interact to impact local retention and anti-tumor efficacy in mice. To extend beyond the empirically testable parameter space, we employ computational tools to predict IL-2’s intratumoral exposure across a spectrum of theoretical sizes (1 kD to 1000 kD) and matrix-binding affinities (micromolar to subnanomolar). Although matrix-binding increases local persistence of intratumorally injected agents as reported previously^29^, we find that tumor exposure can also be improved significantly by increasing molecular size, and maximal exposure is obtained by combining both approaches. Tracking intratumorally injected proteins using positron emission tomography (PET) reveals that increased molecular size not only slows overall diffusive escape from the tumor, as our computational model predicts, but increases the proportion of injected protein halted in potentially viscous regions of the tumor^30^. Collectively, this framework delineates tunable pharmacokinetic features to aid in the engineering of local immunotherapies for maximal anti-tumor effect.

## Results

### Engineering IL-2 fusions of varying size and matrix affinity

**Fig. 1:**
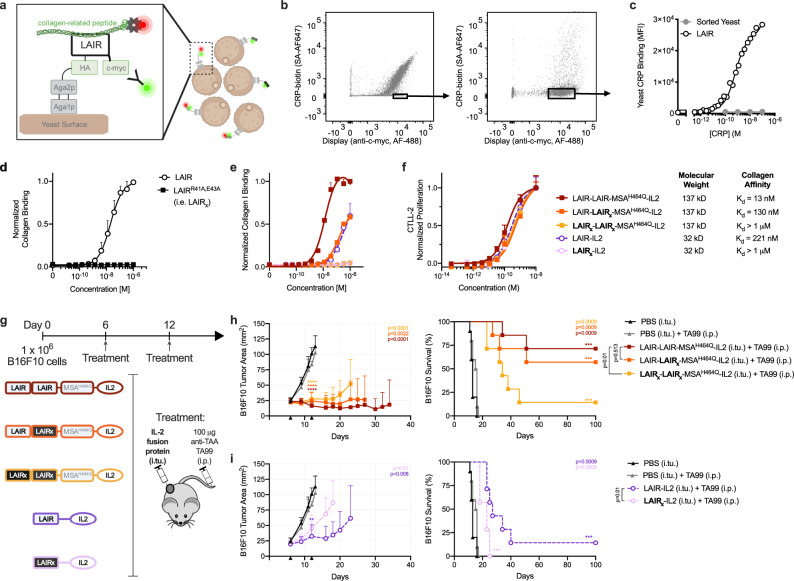
Molecular weight and collagen affinity are key determinants of IL-2 efficacy. **a** Schematic of yeast library displaying mutagenized-LAIR (white) as Aga2 (gray) fusion proteins. LAIR expression was detected using a fluorescent antibody against c-myc epitope (green) and binding to biotinylated collagen-related peptide (CRP) was detected using fluorescent streptavidin (SA-AF647, red). **b** Successive flow assisted cells sorts isolating LAIR mutants with diminished binding to biotinylated CRP. **c** Equilibrium binding titration of biotinylated CRP on enriched yeast from panel **b** (mean + s.d.; *n* = 8) compared to wild-type LAIR-displaying yeast (*n* = 1). Binding was measured by median fluorescence intensity of SA-AF647 by flow cytometry. **d** LAIR and LAIR^R41A E43A^, henceforth LAIR_x_, binding to collagen measured by enzyme-linked immunosorbent assay (ELISA) (mean + s.d., *n* = 6 for LAIR and *n* = 3 for LAIR_x_). **e** Collagen binding affinity (*K*_d_, equilibrium dissociation constant) of IL-2 fusion proteins in small and large molecular weight formats (kD, kilodaltons) measured by ELISA (mean + s.d; *n* = 3 for all groups except *n* = 6 for LAIR-IL2). **f** Dose-dependent proliferation of CTLL-2 cells exposed to IL-2 fusion proteins (mean + s.d.; *n* = 3) **g** Schematic of B16F10 tumor study timeline (top) and treatment components (bottom). Mice were inoculated with 1 × 10^6^ B16F10 cells subcutaneously in the right flank on day 0. Treatments were administered on day 6 and 12. TAA, tumor-associated antigen; i.tu., intratumoral; i.p., intraperitoneal. **h**, **i** Tumor growth (left, mean + s.d.) and survival (right) over time (*n* = 10 mice for the PBS (i.tu.) and PBS (i.tu.) + TA99 (i.p.) groups, and *n* = 7 mice for all other groups). Tumor area is shown until a mouse in the group is euthanized. Statistical significance of each treated group’s tumor area and survival (top right corner) was calculated by using a one-tailed Student’s *t*-test and log-rank Mantel-Cox test, respectively, versus the PBS (i.tu.) + TA99 (i.p.). Other survival comparisons (legend adjacent) were generated by a log-rank Mantel-Cox test. *P < 0.03; **P < 0.002; ***P < 0.0002; ****P < 0.0001; n.s. not significant. Source data for panel **c**–**f** and **h**, **i** are provided in the Source Data file. Panels **a** and **g** created with BioRender.com.

To determine whether an intratumoral immunotherapy’s efficacy depends on its pharmacokinetic features, we sought to create a panel of immunotherapeutic agents varying in molecular weight and collagen-binding affinity. Recently, we and others validated that collagen, an abundant matrix protein, is an effective and tumor-agnostic target for retaining locally administered immunotherapies^29,31–34^. To target immunotherapies to collagen, we used the ectodomain of murine leukocyte-associated immunoglobulin-like receptor-1 (LAIR), an endogenous collagen-binding protein^35^. In order to eliminate LAIR’s collagen affinity while preserving its size, we engineered an inert version of LAIR using yeast surface display^36^. Wild-type LAIR displayed on the surface of yeast specifically bound to collagen-related peptide (CRP), a soluble mimetic of collagen (Fig. 1a and Supplementary Fig. 1a, b)^37^. We generated a library of yeast displaying LAIR mutants (Fig. 1a, b) and sorted clones with diminished binding to CRP (Fig. 1b, c and Supplementary Fig. 2a, b). When expressed recombinantly, these LAIR mutants still retained residual binding to native collagen (Supplementary Fig. 3a–j). Based on the sequences of enriched yeast clones (Supplementary Fig. 2a, b), we then rationally combined mutations to residues deemed critical for CRP binding^38^. Two mutations together, at position 41 (R41A) and position 43 (E43A), eliminated binding to collagen and constitute LAIR_x_, an inert size-matched protein (Fig. 1d).

Like many immunotherapies, IL-2 elicits exposure-dependent anti-tumor effects, making it a model immunomodulator to test the impact of pharmacokinetic properties on therapeutic efficacy. We fused IL-2 to LAIR or LAIR_x_, generating two small-format (32 kD) immunotherapies LAIR-IL2 and LAIR_x_-IL2 (Supplementary Fig. 4a–c) with collagen affinities of 220 nM and >1 μM, respectively (Fig. 1e). Because CRP is an imperfect collagen mimetic, LAIR mutants demonstrating higher-affinity CRP-binding did not exhibit increased affinity to native collagen (Supplementary Fig. 5a–d). Instead, we exploited avidity to increase binding strength by using dimers of LAIR and LAIR_x_. To passively increase molecular weight, we also included a large inert protein, mouse serum albumin (MSA) containing a histidine to glutamine mutation (H464Q) that abrogates neonatal Fc receptor-mediated recycling^39^. The large-format (137 kD) immunotherapies (Supplementary Fig. 4c), LAIR-LAIR-MSA^H464Q^-IL2, LAIR-LAIR_x_-MSA^H464Q^-IL2, and LAIR_x_-LAIR_x_-MSA^H464Q^-IL2 demonstrated collagen affinities of 13 nM, 130 nM, and >1 µM, respectively (Fig. 1e). All IL-2 fusion proteins were equivalently bioactive in inducing T cell proliferation (Fig. 1f).

### Size and matrix affinity of local IL-2 determine response

As a single agent, IL-2 therapy cannot overcome immune resistance barriers posed by the tumor microenvironment. However, we and others have demonstrated previously that IL-2, when combined with a tumor-targeting antibody, can achieve exposure-dependent tumor control mediated by local stimulation of cytotoxic CD8^+^ T and NK cells^29,40–43^. Therefore, we treated mice bearing established, immune-infiltrated (Supplementary Fig. 6), subcutaneous flank B16F10 melanoma tumors with an antibody directed against a tumor-associated antigen tyrosinase-related protein-1 (anti-TYRP-1, or TA99) intraperitoneally and with intratumoral injections of our IL-2 fusion proteins (Fig. 1g). All IL-2 fusion proteins imparted tumor growth delay (Fig. 1h, i); however, treatment with the large-format fusions (Fig. 1h) further slowed tumor growth (*p* = 0.01 for LAIR_x_-LAIR_x_-MSA^H464Q^-IL2 vs. LAIR_x_-IL2) and enhanced survival (*p* = 0.01 for LAIR_x_-LAIR_x_-MSA^H464Q^-IL2 vs. LAIR_x_-IL2; *p* = 0.04 for LAIR-LAIR_x_-MSA^H464Q^-IL2 vs. LAIR-IL2) compared to their small-format counterparts (Fig. 1i). Within each size tier, therapies with collagen binding improved overall survival (Fig. 1h, i). However, the difference in survival after treatment with the two large-format collagen binding variants LAIR-LAIR-MSA^H464Q^-IL2 and LAIR-LAIR_x_-MSA^H464Q^-IL2 of different collagen affinity was apparently not statistically significant (Fig. 1h). Strikingly, the tumor growth delay (*p* = 0.25) and survival benefit (*p* = 0.21) of targeted small-format LAIR-IL2 was equivalent to untargeted large-format LAIR_x_-LAIR_x_-MSA^H464Q^-IL2. Previous work has shown that systemically delivered large-format IL-2 (>70 kD) controls tumors better than wild-type IL-2 (15 kD) due to increased tumor exposure arising from size-based enhancements in circulatory half-life^40^. To compare solely the local effects of LAIR-IL2 and LAIR_x_-LAIR_x_-MSA^H464Q^-IL2, we matched the systemic IL-2 exposure by supplementing local treatments with LAIR_x_-LAIR_x_-MSA^H464Q^-IL2 delivered intraperitoneally (Supplementary Fig. 7a). We found again that the tumor growth delay and survival benefit (Supplementary Fig. 7b, c) of small targeted LAIR-IL2 was indistinguishable from large untargeted LAIR_x_-LAIR_x_-MSA^H464Q^-IL2. Their therapeutic equivalency indicates that, even for therapies injected directly into the tumor, molecular size is very relevant to local exposure.

### Model predicts duration of local treatment activity

To understand and predict how a treatment’s pharmacokinetic properties skew therapeutic efficacy, we recapitulated the kinetic and transport dynamics occurring during and after intratumoral injection into a computational model. During an intratumoral injection, the fluid in the tumor interstitium (i.e. extracellular space between cells in the tumor) is instantaneously displaced by the volume injected. Theoretically, for an injection into a well-mixed tumor interstitium, the volume held-up in the tumor (*V*_holdup_) is related to the tumor’s size (*V*_tumor_) and interstitial void volume fraction (ε_tumor_):

1 $${{{V}}}_{{{{{{\rm{holdup}}}}}}}=({{{V}}}_{{{{{{\rm{tumor}}}}}}})\times {{{{{{\rm{\varepsilon }}}}}}}_{{{{{{\rm{tumor}}}}}}}$$

**Fig. 2:**
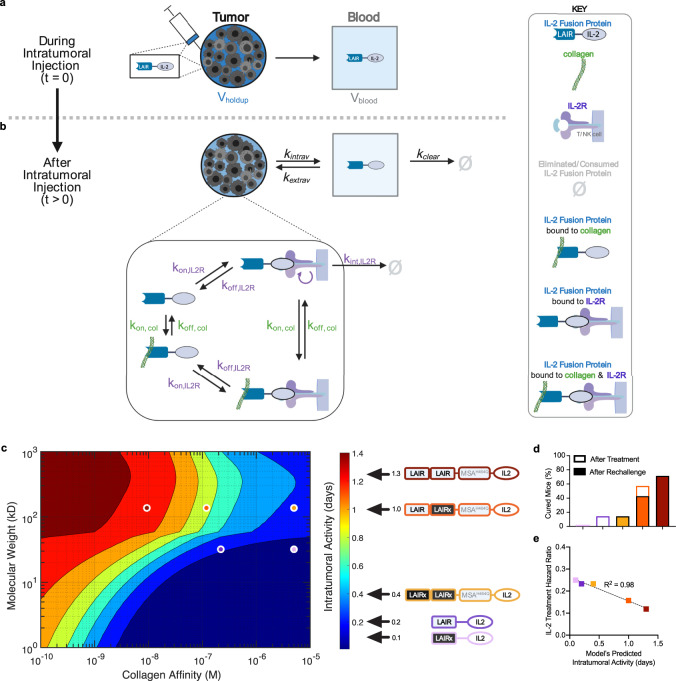
Pharmacokinetic model predicts response to local IL-2 therapy. Model simulating the disposition of intratumorally injected IL-2 collagen-binding fusion protein over time (t). **a** During an intratumoral injection, IL-2 fusion protein is instantaneously transferred from the syringe to the tumor compartment (circle) of volume capacity *V*_holdup_ and blood compartment (square) of volume *V*_blood_ **b** After injection, diffusion of the injected protein between the tumor and blood compartment occurs based on the rate of intravasation (*k*_intrav_) and extravasation (*k*_extrav_). Removal of the injected protein from blood occurs at a rate of clearance (*k*_clear_). Ø indicates removed protein. The italicized rates depend on the molecular weight of the injected protein. Within the tumor interstitium (inset), the injected protein toggles between target-bound states. The injected protein (free or collagen-bound) binds IL-2 receptor (IL-2R) according to rate of association (*k*_on,IL2R_) and dissociation (*k*_off,IL2R_) with IL-2R. When bound to IL-2R, the injected-protein-IL-2R complex is internalized and degraded at the rate of IL-2R internalization (*k*_int,IL2R_). IL-2 signaling increases the concentration of IL-2R over time. The injected protein (free or IL-2R bound) binds collagen according to the rate of association (*k*_on,col_) with collagen and rate of dissociation with collagen (*k*_off,col_). Glossary of the model’s variables, initial conditions, and equations are provided in Supplementary Tables 1–3. **c** Model’s prediction of the intratumoral activity duration of an injected IL-2 fusion protein varying in molecular weight (kD) and collagen affinity (*K*_d_ equilibrium dissociation constant in M units) The circles on the heatmap indicate the model’s prediction for the proteins LAIR-LAIR-MSA^H464Q^-IL2 (red), LAIR-LAIR_x_-MSA^H464Q^-IL2 (orange), LAIR_x_-LAIR_x_-MSA^H464Q^-IL2 (yellow), LAIR-IL2 (purple), and LAIR_x_-IL2 (pink). Each protein’s predicted days of activity is adjacent to color bar. **d** Percentage of mice from Fig. 1h, i surviving 100 days after treatment (outlined box). On day 100, surviving mice were inoculated with 0.1 M B16F10 on the contralateral left flank. The percentage of mice surviving for another 100 days after tumor rechallenge (filled box). **e** Correlation between the model’s predicted intratumoral activity in panel c and hazard ratio for treatments from Fig. 1h, i compared to PBS (i.tu.) + TA99 (i.p.) treated control group. Hazard ratio computed by log-rank method. Source data for panel **d**, **e** are provided in the Source Data file. Panel **a** and **b** created with BioRender.com.

The model assumes that any injected volume not held-up is released immediately into circulation, which is in agreement with observations of systemic dissemination during intratumoral injection^44,45^. (Fig. 2a) After injection, held-up IL-2 fusion protein toggles between four states within the tumor: unbound, bound to collagen, bound to IL-2 receptor (IL-2R), or concurrently bound to both targets (Fig. 2b). Protein-binding interactions are dictated by association (*k*_on_) and dissociation (*k*_off_) rate constants, and the concentration of a complex and its constituents. The model includes two drug removal processes: (1) consumption by IL-2R^+^ cells in the tumor and (2) clearance from blood. Consumption was captured using the internalization rate (*k*_inter_) of the IL-2:IL-2R complex. Since IL-2 induces NK and T cell proliferation, we incorporated a logistic growth function to simulate the increase in IL-2R in response to IL-2 consumption. Since uniformly high interstitial pressures within tumors are thought to eliminate fluid pressure gradients across vessel walls and interstitium, our model of intratumoral drug transport describes diffusive, not convective, processes^18,46^. Protein intravasation (*k*_intrav_) and extravasation (*k*_extrav_) are captured by diffusive transport across a two-pore model of the intratumoral capillaries with a capillary permeability that depends on the protein’s molecular weight, a relationship validated by Schmidt et al.^16^. Within the blood, the rate of plasma clearance (*k*_clear_) uses another validated empirical relationship also dependent on the protein’s molecular weight^16^. Due to the lack of functional lymphatic vessels observed in tumors, transport into the lymphatic system is not modeled here^47,48^. We translated the relationships that define kinetic, transport, and cellular dynamics into a system of ordinary differential equations (ODEs) (Supplementary Tables 1–3, the code generated is also publicly available^49^.) Solving the ODEs yields the total extracellular concentration of the injected IL-2 fusion protein, target-bound and freely diffusing, in the tumor over time. (Supplementary Fig. 8a) To correlate the model output with biological activity, we evaluated the tumor concentration of locally-injected IL-2 fusion protein  ([Intratumoral IL-2 Fusion Protein]) over time using this relationship:

2 $${{{{{\mathrm{Fractional}}}}}}\,{{{{{\mathrm{Activity}}}}}}=\frac{1}{1+\frac{{{{{{\mathrm{E}}}}}}{{{{{{\mathrm{C}}}}}}}_{50,{{{{{\mathrm{IL}}}}}}2}}{[{{{{{\mathrm{Intratumoral}}}}}}\,{{{{{\mathrm{IL-2}}}}}}\,{{{{{\mathrm{Fusion}}}}}}\,{{{{{\mathrm{Protein}}}}}}]}}$$

where EC_50,IL2_, the IL-2:IL-2R half maximal effective concentration, is 2.4 × 10^−7^ M (Eq. 2)^50^. When the intratumoral injected IL-2 concentration far exceeds EC_50,IL2_, the fractional activity approaches 1. However, when the concentration equals EC_50,IL2_, thereby eliciting a half-maximal response, the fractional activity is 0.5. (Supplementary Fig. 8b) The area-under-curve (AUC) of the fractional activity over time estimates how many days after local administration the injected protein is active within the tumor. (Supplementary Fig. 8b)

By inputting a spectrum of collagen affinities and molecular weights into the model, we generated a heat map relating the size and collagen affinity of injected immunotherapies to their duration of activity in the tumor. (Fig. 2c). The activity duration predicted by the model is 1.33 days (~32 h) for LAIR-LAIR-MSA^H464Q^-IL2, 0.99 days (~24 h) for LAIR-LAIR_x_-MSA^H464Q^-IL2, 0.44 days (~11 h) for LAIR_x_-LAIR_x_-MSA^H464Q^-IL2, 0.24 days (~6 h) for LAIR-IL2, and 0.13 days (~3 h) for LAIR_x_-IL2 (Supplementary Fig. 8b and Fig. 2c) The model’s rank-order of treatment activity duration aligns exactly with their elicited in vivo survival and immunological memory (Fig. 2d) as well as the treatment’s hazard ratio (Fig. 2e).

The primary in silico sink for locally injected IL-2 fusion proteins was clearance from circulation and not uptake by T cells, in part due to the low initial abundance of IL-2R+ cells in our poorly inflamed B16F10 tumor model^51^. (Supplementary Fig. 9a) Despite significant drug dissemination to blood both during and after the simulated injection (Supplementary Fig. 9b), the predicted total systemic exposure remained orders-of-magnitude lower than tumor exposure (Supplementary Fig. 9c). Based on a sensitivity analysis of the model’s rates and initial conditions, the parameters-in addition to collagen affinity and molecular weight- that perturb the output tumor exposure are: tumor collagen concentration, the initial tumor concentration of injected protein, and collagen’s half-life (Supplementary Fig. 10). Concentration estimates were derived from tumor hydroxyproline content measurements^52^ (Supplementary Fig. 11a and Supplementary Table 2) and from calculation of injection hold-up with reported values for tumor void volume fraction^16,53,54^. (Supplementary Table 1) Collagen turnover, a slow process facilitated by tumor macrophages, led to negligible measured drug degradation (Supplementary Fig. 11b–e) and thus is not simulated in the model^55^. Nonetheless, even large variation in our choices for concentrations and half-life still corroborate the model’s finding: tumor exposure is indeed impacted heavily by molecular weight and matrix targeting of proteins that are injected intratumorally.

### Large proteins are predisposed to tumor matrix entrapment

**Fig. 3:**
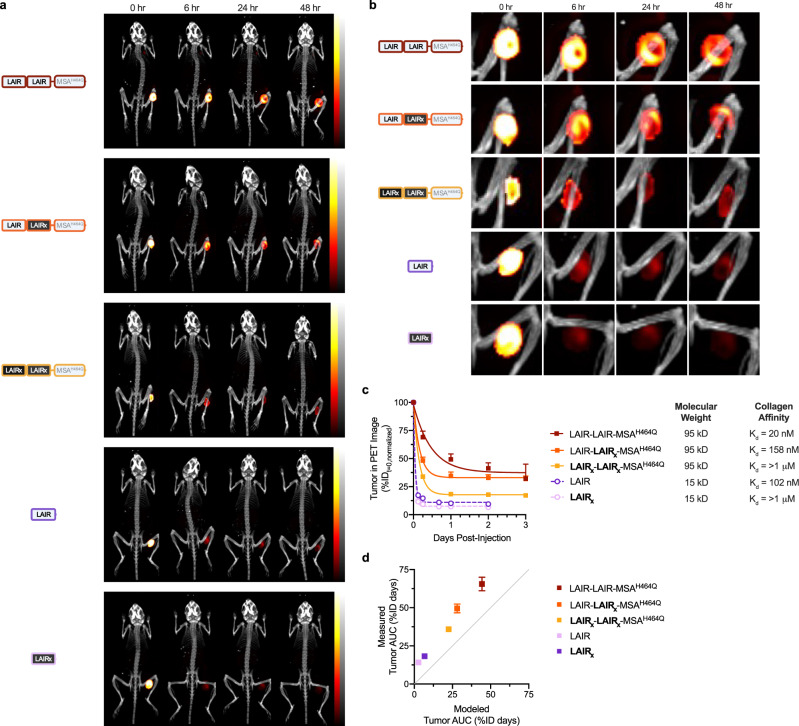
Locally injected protein retention tuned by size and collagen affinity. **a** Representative isotope decay- and PVC-corrected whole body PET/CT 3D intensity projections of B16F10 tumor-bearing mice at 0, 6, 24, and 48 h after intratumoral injection of ^89^Zr-labeled fusion proteins LAIR-LAIR-MSA^H464Q^, LAIR-LAIR_x_-MSA^H464Q^, LAIR_x_-LAIR_x_-MSA^H464Q^, LAIR, and LAIR_x_. CT image (color bar in grayscale, Hounsfield units) is used for anatomical reference. PET emission (red-yellow color scale from 0–100 %ID) is normalized for all images to the maximum activity intensity at time 0 to allow inter- and intra-group comparisons. **b** Injected B16F10 tumors in whole body PET/CT 3D projections of panel **a** are enlarged to visualize injected proteins local distribution. **c** Quantification of intratumoral activity after injection of ^89^Zr-labeled LAIR-LAIR-MSA^H464Q^ (mean + s.d., *n* = 5), LAIR-LAIR_x_-MSA^H464Q^ (mean + s.d., *n* = 5), LAIR_x_-LAIR_x_-MSA^H464Q^ (mean + s.d., *n* = 4), LAIR (mean + s.d., *n* = 4), or LAIR_x_ (mean + s.d., *n* = 4) in corrected PET images. Fusion protein molecular weight (kD, kilodaltons) and affinity (*K*_d_, equilibrium dissociation constant) to collagen type I, measured in Supplementary Fig. 12, displayed adjacent to legend. **d** Comparison of tumor exposure of fusion proteins predicted by the model and measured in vivo (mean ± s.d., *n* = 4–5). Tumor exposure is calculated as the area under the tumor concentration of the injected protein-time curve for a day after injection. Panel **c**, **d** are provided in the Source Data file. ID injected-dose, AUC area-under-the-curve.

To validate the model’s drug disposition, we tracked ^89^Zr (78-hour half-life) labeled proteins after injection into B16F10 tumors using PET/CT-imaging. To minimize the contribution of cell-residualized radionuclide to total intratumoral activity, we removed IL-2 from our fusion proteins. (Supplementary Fig. 12a–c) For consistency, we also adjusted the protein concentration after radioisotope labeling to ensure each tumor was injected with exactly 0.1 nmol protein in 20 μL (Supplementary Table 1). Mice were imaged at multiple time points after injection (Fig. 3a, b and Supplementary Fig. 13a–e). In contrast to systemically-administered agents, intratumorally injected proteins were concentrated more at the tumor than any other organ. Partial volume correction (PVC) and isotope-decay correction of the PET images enabled accurate quantification of protein in the tumor after injection (Fig. 3c and Supplementary Fig. 13f). Notably, injected tumors initially held-up only 30% of the injected dose (I.D.) due to the injected volume (20 μL) exceeding the B16F10 hold-up volume (~6.6 μL) (Supplementary Fig. 14a). Interestingly, the volume held-up was independent of tumor size for the range (20–80 mm^3^) evaluated in our PET study. (Supplementary Fig. 14a). Very large B16F10 tumors (200–300 mm^3^) that better approximate the size of human tumors, however, hold up 66% of our injected-dose which is still only ~13 μL, suggesting that the tumor interstitium is not as rapidly well-mixed as previously thought (Supplementary Fig. 14a). As a result of injecting volume in excess of the tumor’s capacity, we detected immediate protein accumulation in peripheral organs, albeit at concentrations significantly lower than at the tumor and difficult to resolve by PET imaging. (Supplementary Fig. 14b–f)

The amount of intratumorally retained protein decreased more rapidly for the small 15 kD proteins LAIR (*t*_1/2_ = 48 min) and LAIR_x_ (*t*_1/2_ = 40 min) compared to their larger 95 kD equivalent-affinity counterparts LAIR-LAIR_x_-MSA^H464Q^ (*t*_1/2_ = 2.8 h) and LAIR_x_-LAIR_x_-MSA^H464Q^ (*t*_1/2_ = 2.5 h), respectively (Fig. 3c). LAIR-LAIR-MSA^H464Q^ demonstrated the slowest rate of escape from the tumor (*t*_1/2_ = 7.9 h). (Fig. 3c). The model’s predicted rate of tumor escape for these proteins agrees with their observed diffusive escape. (Supplementary Fig. 15a–c). However, the model consistently underestimated their absolute tumor exposure, due to a small fraction of all injected-proteins persisting in the tumor, even at the experimental endpoint. (Fig. 3d and Supplementary Fig. 16) Maximum intensity projections of each individual PET image showed that the persisting proteins were localized to the tumor margin (Supplementary Fig. 17). Quantitative analysis of injected tumors revealed <0.001% of the injected dose residualized inside cells, persuading us that the contribution of non-specific protein catabolism is negligent. (Supplementary Fig. 11b–e). The margins of a B16F10 tumor is matrix-rich, particularly in collagen^29^; however, even inert LAIR_x_ and LAIR_x_-LAIR_x_-MSA^H464Q^ accumulated in this region (Supplementary Fig. 17). The matrix has been shown to considerably slow the diffusion^30^ and convection^56^ of proteins, rendering them entrapped. (Fig. 3b). We observed that the entrapped fraction differed as a function of both molecular weight and collagen binding. The large collagen-binding proteins LAIR-LAIR-MSA^H464Q^ and LAIR-LAIR_x_-MSA^H464Q^ plateaued similarly to 37.3% and 33.0% of the held-up dose, respectively (Fig. 3c). The large inert protein LAIR_x_-LAIR_x_-MSA^H464Q^ plateaued to 17.8% of the held-up dose (Fig. 3c). Amongst the small proteins, 11.1% of held-up LAIR endured compared to only 7.5% of LAIR_x_ (Fig. 3c). Our PET image observations, validated by gamma-counter measurements on excised organs (Supplementary Fig. 16), are consistent with a previous report that proteins within a tumor can become entrapped in viscous matrix regions and that large proteins are more susceptible to entrapment than small proteins^30^. However, this underappreciated dynamic is not recapitulated by the current model.

### Contribution of size to exposure outweighs affinity to FN-EIIIB

We sought to evaluate whether the role of size and binding affinity extends to IL-2 fusion proteins targeting less abundant (Supplementary Fig. 18a) yet tumor-specific matrix proteins. We fused IL-2 to the 15 kD nanobody NJB2, previously shown to bind alternatively spliced extra-domain B (EIIIB) domain of fibronectin (FN-EIIIB), a tumor-specific glycoprotein, with nanomolar affinity. (Supplementary Fig. 18b)^57,58^ To generate an inert size-matched comparator, we fused IL-2 to NJT6, a nanobody that lacks a murine target. To increase the molecular weight while maintaining affinity, MSA^H464Q^ was incorporated between the nanobody and IL-2 (Supplementary Fig. 18b). The 32 kD small format, NJB2-IL2 and NJT6-IL2, and 98 kD large format, NJB2-MSA^H464Q^-IL2 and NJT6-MSA^H464Q^-IL2, equivalently activated a T cell line in culture (Supplementary Fig. 18c).

**Fig. 4:**
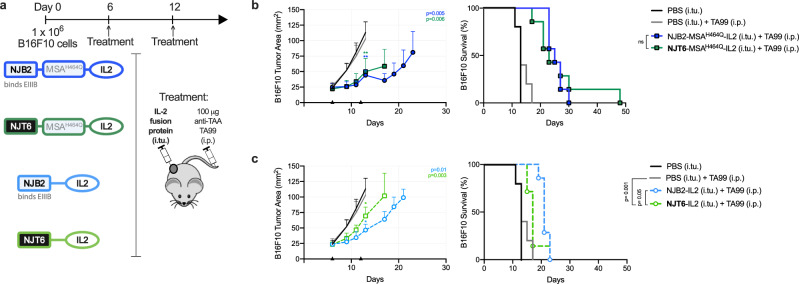
Contribution of size to exposure outweighs target-binding affinity for less abundant target EIIIB. **a** Schematic of the B16F10 tumor immunotherapy study timeline (top) and treatment components (bottom). Mice were inoculated with 1 × 10^6^ B16F10 cells subcutaneously in the right flank on day 0. Six days after tumor implantation, treatments were initiated following the timeline. (*n* = 5 mice for the PBS (i.tu.) and PBS (i.tu.) + TA99 (i.p.) groups, and *n* = 7 mice for all other groups). TAA tumor-associated antigen, i.tu. intratumoral, i.p. intraperitoneal. **b**, **c** Tumor growth over time (left, mean + s.d.) and survival (right) of the indicated groups. Tumor area for each group is shown until a mouse in the group is euthanized due to tumor burden. Statistical significance of each treated group’s tumor area (displayed on top right corner) was calculated by using a one-tailed Student’s *t*-test versus the PBS (i.tu.) + TA99 (i.p.) group on day 11. Comparisons for each treated group’s survival was calculated by using a log-rank Mantel-Cox test. *P < 0.03; **P < 0.002; ***P < 0.0002; ****P < 0.0001; n.s. not significant. Source data for panel **b**, **c** are provided in the Source Data file. Panel **a** was created with BioRender.com.

We treated mice bearing established subcutaneous flank B16F10 tumors intraperitoneally with TA99 and intratumorally with our IL-2 nanobody panel (Fig. 4a). Consistent with earlier observations, large-format IL-2 delayed tumor outgrowth more than small-format IL-2. (Figs. 1g and 4b, c) When comparing equivalent affinity constructs, increasing molecular weight improves the overall survival efficacy: NJB2-MSA^H464Q^-IL2 v. NJB2-IL2 (*p* = 0.003) and NJT6-MSA^H464Q^-IL2 v. NJT6-IL2 (*p* = 0.03). However, the EIIIB-targeting only improved the treatment efficacy for the small format NJB2-IL2 over NJT6-IL2 (*p* = 0.05) and not the large-format NJB2-MSA^H464Q^-IL2 vs. NJT6-MSA^H464Q^-IL2 (*p* = 0.92). In agreement with earlier observation that the benefit of size can outweigh the benefit of targeting, the survival with large untargeted NJT6-MSA^H464Q^-IL2 was identical (*p* = 0.13) to small targeted NJB2-IL2. The pharmacokinetics of locally injected EIIIB-targeting IL-2 fusion proteins, particularly their molecular weight, dictates their overall effectiveness.

## Discussion

One purpose of intratumoral administration is to increase the local bioavailability of a therapy (the other purpose being to minimize toxic systemic exposure). Here we explore how the pharmacokinetic properties of locally injected biologics, specifically molecular size and matrix-binding affinity, can be tuned to maximize tumor retention and thereby boost anti-tumor responses in mice.

Local administration is a seriously considered option for potent immuno-modulators. Several dose-limited protein therapies are currently in trials as intratumoral therapies: OX40 agonist antibody (NCT03831295), CD137 agonist antibody (NCT03792724), bispecific antibody MDX-447 (NCT00005813), anti-PD-1 and anti-CTLA-4 antibodies (NCT03058289), and tumor-targeted IL-2 and TNFα (NCT04362722). The absence of a fundamental pharmacokinetic framework for intratumorally injected agent biodistribution restrains their design optimization. Direct intratumoral injection of an immunotherapy ensures access to immune cells residing within the tumor microenvironment. But since local treatment can act as an in situ vaccine, a robust influx of target immune cells may not occur for several hours to days after treatment. Prolonging tumor residence of injected agents to guarantee productive engagement with desired immune cells is critical to safely maximizing efficacy. Furthermore, to sustain biological effect, the local drug concentration must remain within its therapeutic window. The product of tumor drug concentration and duration of bioavailability equates to local drug exposure. Intratumoral drug exposure has predicted overall survival of humans treated with checkpoint blockade^59,60^, and of mice treated with IL-2, as demonstrated in the present work.

Using computational and experimental methodologies, evaluating different IL-2 fusion proteins highlighted the decisive role of size and matrix binding on exposure-driven efficacy (Figs. 1 and 4). To our surprise, modest differences in molecular weight of IL-2 fusion proteins significantly impacts their efficacy. Biodistribution studies on intratumorally injected dextrans^12^ and liposomes^13^ have found that a critical diameter between 30–50 nm and 100–200 nm, respectively, marks a major inflection in tumor retention. LAIR (~3 nm) and LAIR-LAIR-MSA (~7 nm) are comparatively smaller and poorly-retained yet we find their size-based differences in retention are nevertheless therapeutically meaningful. It is well established that size controls a molecule’s diffusivity within the tumor interstitium and its vascular permeability, thus larger proteins escape the tumor more slowly (Fig. 3b). However, we observed that intratumorally injected proteins are subject to an additional size-based phenomenon: entrapment (Fig. 3c). Alexandrakis et al. first identified entrapment when reporting that an intratumorally injected protein exhibits two interstitial diffusivities and that protein partitioning to “fast” (~10^−7^ cm s^−1^) and “slow” (~10^−9^ cm s^−1^) diffusing states depends on its molecular weight. They found that 20% of bovine serum albumin (69 kD) injected into a melanoma tumor model partitions into a slow diffusing state, appearing immobilized. Consistent with this measurement, we observed 18% of LAIR_x_-LAIR_x_-MSA^H464Q^ (97 kD) and 8% of LAIR_x_ (15 kD) appear entrapped in B16F10 melanoma tumors (Fig. 3c). PET imaging revealed that our injected proteins become entrapped in the tumor periphery, an area rich in collagen matrix and responsible for slowing protein diffusivity^29^ (Fig. 3b and Supplementary Fig. 17). Size-based entrapment could partially explain why larger IL-2 fusions imparted better tumor growth delay and survival benefit than smaller IL-2 fusions. However, we find that the small and large protein versions persist at intratumoral concentrations of 50 nM and 200 nM, respectively, both exceeding the EC50 for IL-2Rɑβɣ activation (Supplementary Fig. 16). Further studies into how entrapment in viscous tumor regions impacts cytokine bioavailability are warranted.

Tuning tumor exposure of locally injected proteins can also be accomplished by functionalization with a tumor-localization domain. To date, researchers have appended local immuno-modulators with tumor-localization domains that bind acellular matrix proteins (collagen^29,31–34^, fibronectin splice variants^26^, tenascin C^61^, etc.) and cellular targets^27,28^. We find that successful localization depends on target abundance. FN containing EIIIB and collagen are both long-lived matrix proteins; however, FN containing EIIIB is far less abundant than collagen in B16F10 tumors (Supplementary Fig. 18a)^58,62^. As a result, we observe that the survival elicited by NJB2-MSA^H464Q^-IL2 (*K*_d,EIIIB_ 1 nM) treatment is inferior to that of LAIR-LAIR-MSA^H464Q^-IL2 (*K*_d,collagen_ 13 nM). Our model predicts that tumor cell targets of similar abundance to collagen (with an expression of ~10^5^–10^6^ receptors per cell) may also impart localization. Conceivably, achieving productive tumor localization with a cellular target will require balancing binding affinity and target turnover in order to minimize target-mediated drug degradation. The modeling approach described enables follow up analyses on the extent drug exposure is affected by alternative targets (varying in abundance and turnover) and even other pharmacokinetic properties (e.g. charge, stability, and hydrophobicity). With minor modification, the existing model can predict the retention of other immune modulators. In a vast parameter space, employing computational tools in drug design as done here can reveal the principles and major criteria that best drive improvements in efficacy.

Depending on the immune agonist and its mechanism of action, eliciting a desired biological effect may depend on drug exposure in the tumor and/or other lymphoid tissues such as the tumor-draining lymph node (tdLN). For IL-2 therapy of a single lesion, tumor exposure alone sufficiently predicts response. However, for co-stimulatory or co-inhibitory molecules, tdLN exposure may be a better indicator of effectiveness^63^. Fortunately, for biologics, prolonged tissue residence enhances lymphatic drainage^64^. Therefore, extending tumor interstitium persistence after local injection may be a simple way to ensure and augment drainage of agents to the functional lymphatic vessels found at the tumor periphery^65^. Our model may still provide a general ranking for proteins of different sizes whose actions depend on tdLN exposure, and not tumor exposure directly. Alternatively, transport to the tdLN can be empirically defined, using relationships established for subcutaneously injected drugs^66^, and appended to the model framework.

Importantly, prolonged tumor exposure will not necessarily translate to enhanced efficacy for all immunotherapies^67^. Some cytokines that coordinate anti-tumor immunity, such as type I^68^ and type II^69^ interferons, elicit effects (such as upregulation of PD-L1/L2^70^, T cell activation induced cell death^71^, etc.) detrimental to anti-tumor immunity at prolonged high tumor concentrations. The exposure-response relationship for other cytokines may also hook at high levels of exposure. The LAIR and LAIR_x_ fusion proteins and computational model described here afford the opportunity to probe for the optimal signal duration of various cytokine therapies.

By simplifying the tumor into a single compartment, our model does not address how spatial heterogeneity in antigen distribution, hydraulic conductivity, and interstitial fluid pressure (IFP) within different tumors impacts the exposure and efficacy of injected drugs. It is widely accepted that uniformly elevated IFP inside tumors inhibits transvascular and interstitial convection rendering diffusion the dominant mode of transport within tumors^18,46^ - just as we have modeled. However, the pressure drop at the interface between tumor and normal tissue can create an outward seeping flow at the tumor margin. Concurrently, the low hydraulic conductivity of the matrix-rich interface also can impede convective fluid flow^56^. The interplay of these dynamics results in intratumorally injected proteins stalled in the margin of B16F10 tumors. (Supplementary Fig. 17) For tumor models displaying different matrix composition, solid stress, size, etc. their combined transport dynamics may result in a different localization pattern for intratumorally injected proteins^72^. Understanding and integrating all spatial dynamics that describe intratumoral micro-pharmacokinetics, although experimentally and computationally intensive, can refine the proposed model. The impact of intratumoral drug distribution is a critical issue that contributes to varied local pharmacodynamic responses to small molecule inhibitors^73^ and antibody-drug conjugates^74,75^.

Once local drug candidates are identified, the procedures to locally administer them are critical to their successful translation. The optimal injection technique that maximizes intratumoral drug distribution and initial retention is unknown. We find that B16F10 tumors accommodate only ~6.6–13.3 μL of injected volume, implying that our injection only accessed a tumor subregion. Studies in this tumor model typically test volumes from 10–30 µL, and thus a bulk of a locally injected dose is actually systemically injected. Injecting volumes in excess of tumor capacity, a common practice in preclinical mouse models, occurs in the clinical setting as well. Investigational local therapies often follow guidance established by the first intratumoral oncolytic viral therapy talimogene laherparepvec (T-VEC, trademarked Imlygic) which advises injecting between a tenth to as much as 30-fold greater fluid volumes than a tumor can theoretically hold. As a result, systemic dissemination during intratumoral injection is not uncommon and has been observed for agents as small as tracers and as large as viral vectors^45,76^. Whether some drug leakage from an injected lesion is necessary to engender a robust systemic immunological response has not been explored. Clinically, the consequences of imperfect local retention are diminished efficacy and increased toxicity due to unintended systemic exposure of potent drugs. In an assessment of intratumoral injections, severe (grade ≥ 3) immune-related systemic toxic effects occurred in 4% of T-VEC injections and 2% of investigational local therapies due to drug leakage^77^. Standardized instructions for intratumoral injection, aimed at reducing immediate systemic exposure while maximizing tumor coverage, are much needed. Currently, clinical protocols suggest scaling intratumoral injection volume by lesion size;^1,78^ but as we observed in mice, larger lesions may accommodate the same initial hold-up volume from a single injection as small lesions – a poorly understood phenomenon. Defining the optimal injection number, volume, rate, and pressure as a function of tumor and drug properties is needed to improve delivery. While generating guidelines based on observations in syngeneic mouse models – as we have done here – is helpful, their validation in tumors of clinically-relevant size and tissue heterogeneity is still required.

Clinical outcomes of local immunotherapies depend on both their administration and local pharmacokinetics. To the latter end, we have outlined here a foundational modeling framework establishing the pharmacokinetic underpinnings of local IL-2 therapy efficacy in mice. Our analysis presents a step towards the critical evaluation of locally injected biologics, which should help the growing field of intratumoral pharmacology rationally design the properties and dosing regimens of future local immunotherapies.

## Methods

### Mice

All animal work complies with regulations established by Massachusetts Institute of Technology Committee of Animal Care (CAC) and Division of Comparative Medicine (DCM) under a protocol approved by the Institional Animal Care and Use Committee (IACUC) in accordance with federal, state, and local guidelines. Female B6 mice (C57BL/6NTac) between 6–8 weeks of age were purchased from Taconic.

### Cells

B16F10 cells (ATCC, CRL-6475) were cultured in Dulbecco’s modified Eagle’s medium (ATCC) supplemented with 10% FBS (Thermo Fisher Scientific). HEK293-F (Life Technologies, R79007) were cultured in Freestyle medium (Life Technologies). CTLL-2 cells (ATCC, TIB-214) were cultured in RPMI-1640 (ATCC) with 10% FBS, 10% T cell culture supplement with concanavalin A (T-STIM with ConA, Corning), 20 mM HEPES, 1 mM sodium pyruvate, 0.05 mM β-mercaptoethanol (Life Technologies), 100 units/ml penicillin (Life Technologies), 100 µg/ml streptomycin (Life Technologies), 2 mM l-alanyl,-l-glutamine (Life Technologies), and 1x minimal essential medium (Corning) non-essential amino acids. All cells and cell assays were maintained at 37 °C and 5% CO^2^. All cell lines were tested for mycoplasma contamination, and none tested positive.

### In vivo tumor survival studies and treatment

For inoculation of B16F10, 10^6^ cells resuspended in 50 μL of sterile PBS were injected subcutaneously into the right flanks of C57BL/6 female mice.

For all tumors studies, we initiated treatment once tumors were established, defined as being palpable or measuring ~25 mm^2^ in area and thus amenable to an intratumoral injection. Treatments were administered to tumor-bearing mice six and twelve days after tumor inoculation. TA99 was administered at 100 μg per dose intraperitoneally (i.p.) in 100 μL of sterile PBS. Intratumoral treatments into the subcutaneous flank tumors were administered in 20 μL of sterile PBS. For all intratumoral IL-2 treatments (in Figs. 1 and 4), an equivalent of 0.1 nmol per dose in a total 20 μL volume of PBS was injected directly into tumors. For all systemic IL-2 treatments (in Supplementary Fig. 7), an equivalent of 0.1 nmol per dose in a total of 50 μL of PBS was injected intraperitoneally. For all intratumoral radiolabeled proteins treatments (in Fig. 3), an equivalent of 0.1 nmol per dose in a total 20 μL volume of PBS was injected directly into tumors.

Mice were randomized into treatment groups immediately before the first treatment. Tumor size was measured as an area (longest dimension × perpendicular dimension) three times weekly, and mice were euthanized when tumor area exceeded 100 mm^2^ or at the end of a predefined experimental endpoint (PET imaging or necropsy). Mice that rejected tumors were re-challenged with 10^5^ matched tumor cells on the left flank at a hundred days after the first tumor inoculum and tumor growth was monitored.

### Yeast surface display of wild-type LAIR and Lumican

LAIR and Lumican encoding gene fragments were amplified by PCR with CloneAmp HiFi PCR-premix (Takara). Both 5′ and 3′ ends of the final PCR-purified product contained 50 base pairs of homology with the ends of the NheI/BamHI-linearized pCTCON2 vector, thereby facilitating two-piece homologous recombination in yeast. The pCTCON2 vector was prepared for homologous recombination by digestion with SalI-HF, NheI-HF, and BamI-HF (New England Biolabs). The LAIR or Lumican PCR fragments along with the digested pCTCON2 vector were co-transformed into chemically competent yeast using the Frozen-EZ Yeast Transformation II Kit (Zymo Research) and resultant clones were sequence verified prior to experiments.

### Affinity dematuration of LAIR using yeast surface display

A library of yeast displaying LAIR with random amino acid mutations was constructed using typical affinity maturation methods described elsewhere^36,79,80^. Briefly, 30 cycles of error-prone PCR was performed on plasmid DNA containing wild-type LAIR using 2 µM concentration of each 8-oxo-dGTP and dPTP. The PCR-purified fragment encoding mutagenized LAIR was amplified by PCR with CloneAmp HiFi PCR-premix (Takara). Both 5′ and 3′ ends of the final PCR product contained 50 base pairs of homology with the ends of the NheI/BamHi-linearized pCTCON2 vector, thereby facilitating two-piece homologous recombination in yeast. Saccharomyces cerevisiae (yeast) strain EBY100 was transformed by electroporation with the linearized pCTCON2 vector and mutagenized LAIR fragments. The resulting library contained 1 × 10^7^ unique transformants encoding the fusion protein Aga2p- HA tag- (Gly4Ser)3 linker- mutant LAIR- c-MYC tag. For all yeast experiments, yeast were grown in SD-CAA medium (containing 20 g/L d-glucose, 6.7 g/L yeast nitrogen base, 5 g/L casamino acids, 7.4 g/L citric acid monohydrate, 10.4 g/L sodium citrate, pH 4.5) to an optical density at 600 nm (OD600) of 1 (equivalent to 1 × 10^7^ cells/mL) at 30 °C. Yeast surface expression of the mutagenised LAIR was induced in SG-CAA medium (containing 18 g/L galactose, 2 g/L D-glucose, 6.7 g/L yeast nitrogen base, 5 g/L casamino acids, 5.4 g/L Na_2_HPO_4_, 8.6 g/L NaH_2_PO_4_monohydrate, pH 6.0) overnight at 20 °C for cultures with a starting OD600 of 1. All subsequent staining of yeast was performed in PBSA (containing PBS + 0.1% bovine serum albumin). For flow assisted cell sorting and cytometry experiments, PBSA-washed yeast were incubated with the CRP antigen containing an N-terminal biotin (see CRP synthesis method) for 1 h at room temperature, followed by labeling with 1:500 chicken anti-c-MYC (ACMYC, Gallus Immunotech) for 30 min on ice, followed by incubation with 1:200 Streptavidin-Alexa Fluor 647 (S21374, Invitrogen) and 1:200 goat anti-chicken Alexa Fluor 488 (A-11039, Invitrogen) for 30 min on ice. Yeast cells were sorted on a FACSAria III Cell Sorter (BD). Sorted yeast were rescued in SDCAA and their plasmid DNA isolated using Zymoprep Yeast Plasmid Miniprep II Kit (Zymo Research). Isolated plasmid DNA was transformed into Stellar Competent Cells (Takara Bio Inc.), yielding several colonies that were sequenced to track the mutations converging after each sort.

### Collagen related peptide synthesis and cross-linking

The synthesis of collagen-related peptide (CRP) is described previously^81^. Briefly, a peptide containing an N-terminal biotin, the following l-amino acids GCO(GPO)_10_GCOG (where O is hydroxyproline) and a C-terminal carboxamide (CONH_2_) was generated by solid phase synthesis. Peptide cleaved from the resin was further purified by reversed-phase high performance liquid chromatography and lyophilized. The weight of the peptide was assumed to be 75% of the mass, the remainder being water/trifluoroacetic acid salts. The CRP peptide used in all yeast display experiments was cross-linked into its most stable trimeric form with *N*-succinimidyl 3-(2-pyridyldithio)propionate (SPDP), which cross-links both cysteine and free-amino acid groups. Briefly, 1.5 mM solution of lyophilized CRP (3531 Da) was prepared in 0.1 M NaHCO_3_ and incubated with 0.5 molar-equivalent of SPDP (from a 15.6 mg/mL stock in dry ethanol) under nitrogen flow for 1 h. The solution was dialyzed in 1–2 L of 10 mM acetic acid at 4 °C for 2 h twice and overnight once. Assuming no losses, the final concentration of cross-linked CRP was the starting molar quantity divided by the final solution volume.

### Cloning

Codon-optimized DNA sequence encoding the ectodomain of murine LAIR-1 was purchased as a GeneArt string fragment (Thermo Fisher). This fragment was cloned by In-Fusion (Clontech) using customized primers (IDT) into the gWIZ vector (Gelantis) to generate these His-tagged proteins: LAIR-his, LAIR-LAIR-MSA^H464Q^-his, LAIR-IL2-his, and LAIR-LAIR-MSA^H464Q^-IL2-his. LAIR_x_ was generated from LAIR by site-directed mutagenesis of an arginine to alanine at position 41 (R41A) and a glutamic acid to alanine at position 43 (E43A). LAIR_x_ containing His-tagged proteins were generated: LAIR_x_-his, LAIR-LAIR_x_-MSA^H464Q^-his, LAIR_x_-LAIR_x_-MSA^H464Q^-his, LAIR_x_-IL2-his, LAIR-LAIR_x_-MSA^H464Q^-IL2-his, and LAIR_x_-LAIR_x_-MSA^H464Q^-IL2-his.

Nanobody (NJB2 and NJT6) fusion proteins were also generated by In-Fusion cloning (Clontech) of the nanobody fragments into the gWIZ vector. Sequences for all fusion proteins are detailed in Supplementary Table 4. Plasmid DNA encoding each fusion protein was transformed and amplified in Stellar Competent Cells (Takara Bio Inc.) and subsequently purified using NucleoBond Xtra Midi EF endotoxin-free midi-prep kit (Takara Bio Inc.).

### Protein production and purification

Suspension HEK293 cells were transfected with sterile-filtered plasmid DNA using polyethylenimine in OptiPro serum-free medium (Thermo Fisher). TA99 was purified using rProtein A Sepharose Fast Flow resin (GE Healthcare) as previously described^40^. His-tagged proteins were isolated from HEK293 supernatant using TALON Metal Affinity Resin (Takara Bio Inc.). Some cytokine-fusion proteins were then further purified by size exclusion chromatography using a HiLoad 16/600 Superdex 200 pg column on an ÄKTA FPLC system (GE Healthcare) that had been pretreated overnight with 1 M NaOH to remove endotoxin and subsequently equilibrated in sterile PBS (Corning). After purification, all proteins were buffer exchanged into sterile PBS (Corning), 0.2 μm sterile-filtered (Pall Corporation), and confirmed to contain minimal endotoxin (<0.1 EU per injection) using a chromogenic LAL assay (Lonza). To confirm their molecular weights, proteins were run alongside a Novex Prestained Sharp Protein Ladder on a 4–12% NuPAGE Bis-Tris protein gel (Life Technologies) with 1% MES running buffer. All proteins were stored at −80 °C, but before therapeutic injection or in vitro assessment, cytokine fusion proteins were thawed on ice.

### Tumor collagen content estimation (hydroxyproline quantification)

Tumor collagen content was extrapolated from their hydroxyproline content, measured using a hydroxyproline quantification kit (MAK008-1KT, Sigma Aldrich) according to manufacturer’s instructions (see details in Supplementary Table 2). Briefly, a standard curve was prepared by diluting 10 µL of the 1 mg/mL hydroxyproline standard solution in 90 µL of water to prepare a 0.1 mg/mL standard solution. Then 0, 2, 4, 6, 8, and 10 µL of the 0.1 mg/mL standard solution were added to a 96-well plate generating a 0 (blank,) 0.2, 0.4, 0.6, 0.8, and 1 μg/well standard. To prepare B16F10 tumor samples, excised tumors were weighed, incubated in 1 mL of water containing 1 mg/mL collagenase and dispase (Roche) and 20 μg/mL DNAse I (Roche) at 37 °C for 1 h and homogenized with 1.0 mm Silica glass beads filled tubes (BeadBug, Sigma Aldrich) in a Mini-Beadbeater-16 (Bio Spec Products Inc). Then 100 µL of each tumor homogenate was hydrolyzed in 100 µL of 12 M hydrochloric acid in pressure-tight polypropylene vials with PTFE-lined caps tightly sealed, wrapped in parafilm and aluminum foil at 120 °C for 3 h. Samples were then mixed and centrifuged at 10,000 × *g* for 3 min. For each sample, 25 µL was transferred to a 96-well plate. The plate containing sample and standard wells was dried in a 60 °C oven overnight. To each well, 100 µL of chloramine T/oxidation buffer mixture was added and incubated at room temperature for 5 min. Then, 100 µL of the diluted DMAB reagent (50 µL of DMAB concentrate and 50 µL of perchloric acid/isopropanol solution) was mixed with each well and incubated at 60 °C for 90 min. Each sample and standard’s absorbance at 550 nm (A550) was measured using an Infinite M1000 microplate reader (Tecan). Samples and standards were corrected for background by subtracting the A550 obtained from the 0 µg standard. The corrected values obtained from the hydroxyproline standards were used to plot a standard curve. Hydroxyproline content from samples was interpolated from the standard curve.

### Pharmacokinetic model

Details and derivations of the mathematical pharmacokinetic model can be found in the Supplementary Tables 1, 2, and 3. The ordinary differential equations were solved with a stiff solver (ode15s) in MATLAB (R2019b, The Mathworks; Natick, MA).

### CTLL-2 proliferation assay

CTLL-2 cells were seeded onto 96-well tissue culture plates at a density of 5000 cells/well in 100 µl of media without T-STIM and without ConA. Cells were cultured with varying concentrations of IL-2 fusion proteins for 48 h. Cell proliferation was determined by WST-1-based colorimetric assay (Roche) according to manufacturer’s instructions. Absorbance at 450 nm (corrected with a reference absorbance at 650 nm) was measured using an Infinite M200 microplate reader (Tecan).

### Collagen enzyme-linked immunosorbent assay

Collagen I (Gibco) coated 96-well plates were blocked at room temperature overnight with PBS with 2% wt/vol bovine serum albumin (BSA) and 0.05% vol/vol Tween-20 and then incubated with various concentrations of LAIR-fusion proteins in PBSTA (PBS with 0.1% wt/vol bovine serum albumin (BSA) and 0.05% vol/vol Tween-20) for 4 h at room temperature. Wells were washed with PBST six times and then incubated with a horseradish peroxidase-conjugated polyclonal anti-6xHis (ab1187, Abcam) at a 1:5000 dilution in PBSTA for 1 h at room temperature. Wells were washed again six times with PBSTA, and then 1-Step Ultra TMB-ELISA Substrate Solution (Thermo Fisher Scientific) was added for 5–10 min followed by 1 M sulfuric acid to stop the chromogenic reaction. Absorbance at 450 nm (corrected with a reference absorbance at 570 nm) was measured on an Infinite M200 microplate reader (Tecan).

### Radiolabeling of proteins

Fusion proteins in PBS, pH adjusted to 8 using 1 M dipotassium phosphate, were labeled with chelator p-SCN-Bn-Deferoxamine (B-705, Macrocyclics) overnight at 4 °C. Excess chelator was removed using PBS-equilibrated PD-10 Columns (GE Healthcare). All proteins were 0.2 μm sterile-filtered (Pall Corporation) prior to radioisotope complexation. All buffers were treated with Chelex 100 Resin (142-1253, BioRad) to prevent iron contamination. Zirconium-89 (or ^89^Zr) was supplied as zirconium oxalate in 1.0 M oxalic acid from the Mallinckrodt Institute of Radiology Cyclotron Facility at Washington University School of Medicine. The zirconium solution was neutralized to pH 7 with half-part-volume of 2 M sodium bicarbonate and one-part-volume of 0.5 M HEPES (e.g. for a 10 μL of zirconium solution, 5 μL of 2 M Na_2_CO_3_, and 15 μL of 0.5 M HEPES was added). For ^89^Zr chelation with DFO-labeled proteins, 3–4 nmol of protein (in a 10–60 μM solution in PBS) was added to 1–3 mCi of neutralized ^89^Zr and incubated at room temperature for 1 h. Free ^89^Zr was separated from ^89^Zr-labeled LAIR-LAIR-MSA^H464Q^, LAIR-LAIR_x_-MSA^H464Q^ and LAIR_x_-LAIR_x_-MSA^H464Q^ using PBS-equilibrated 0.5 mL 7 kD molecular weight cut off Zeba^TM^ Spin Desalting Columns (Thermo Fisher Scientific). Free ^89^Zr was separated from ^89^Zr-labeled LAIR and LAIR_x_ using PBS-equilibrated PD MiniTrap^TM^ G-10 Columns (GE Healthcare). Absorbance at 280 nm for radiolabeled proteins was measured on a Nanodrop^TM^ UV-Vis Spectrophotometer (Thermo Fisher Scientific) and corrected using the tryptophan fluorescence to A280 ratio, extinction coefficient and molecular weight of DFO-labeled proteins to obtain an accurate protein concentration. All radiolabeled proteins were then diluted with PBS a concentration of to 5 μM (0.1 nmol protein per 20 μL). All radiation work was approved by the Radiation Protection Program of the Environmental Health and Safety Department at MIT and was conducted under the Radiation License of Massachusetts Institute of Technology and conforms to all applicable federal, state, and local regulations.

### Positron emission tomography and Computed Tomography Imaging

PET/computed tomography (CT) imaging of intratumorally injected ^89^Zr-labeled proteins was performed by using a G8 PET/CT preclinical small-animal scanner (PerkinElmer, developed by Sofie). Mice were anesthetized with isoflurane (2% mixed with oxygen) and kept warm using controlled heating pads during the PET/CT scan. Mice were imaged with a static PET scan for 10 min followed by a 1.5 min CT scan for anatomical reference. All PET/CT data was reconstructed with a Monte-Carlo based system matrix and an iterative maximum likelihood estimation method (MLEM) algorithm for 50 iterations on 0.456 × 0.456 × 0.456 mm^3^ voxel size with no scatter correction. For radiolabeled LAIR and LAIR_x_, PET/CT scans were performed immediately after injection (0 h) and 3, 6, 17, 24, and 48 h later. For radiolabeled LAIR-LAIR-MSA^H464Q^, LAIR-LAIR_x_-MSA^H464Q^ and LAIR_x_-LAIR_x_-MSA^H464Q^, PET/CT scans were performed immediately after injection (0 h) and 6, 24, 48, and 72 h later. Immediately after each injection, the total injected activity was verified by measuring the difference in syringe activity before and after injection and the activity in the animal using a dose calibrator. After the final PET/CT scan, mice were euthanized and several tissues and organs were collected: blood, right leg femur bone, peritumoral subcutaneous fat, feces, heart, left and right kidneys, large intestine devoid of feces, liver, left and right lung, small intestine devoid of feces, spleen, stomach, tumor-draining lymph node (tdLN), and tumor with proximal skin. These tissues were weighed and measured for radioactivity using a 2480 Wizard2 automatic gamma counter (PerkinElmer).

### PET Image Partial volume correction

Partial volume correction (PVC) of the PET data was performed using PETPVC, an open source toolbox made available by the University College London (github.com/UCL)^82^. Partial volume effect (PVE) is an apparent difference between the calculated and the true activity concentration as a result of limitations in the imaging system’s spatial resolution and spatial sampling. It is particularly pronounced for objects whose size is close to the system’s reconstructed spatial resolution and can result in activity spillover. Due to the spatial resolution of our PET imager and injected tumors’ size, accurate quantification of radiotracer distribution using PET images requires correction of its PVE. PVC algorithms routinely implemented in neuroimaging to quantify bright and small structures in PET images of the brain can be extended to tumor-imaging^83^. Interregional PVC was performed using region-based voxel-wise correction (RBV) in conjunction with the Labbé approach. Intraregional PVC was performed with 10 iterations of the van Cittert (VC) deconvolution algorithm using the scanner’s point-spread-function (x, y, z) of 1.4 mm × 1.4 mm × 1.6 mm. All these corrections were implemented using PET PVC toolbox’s petvc function.

Briefly, to prepare data for PVC, a tissue classification map or mask was generated in VivoQuant by segmenting tissues of interest (tumor, tumor-draining lymph node, liver or kidney and bladder) based on each excised organ’s volume. The VivoQuant DICOM mask was converted into a 32-bit 3D parcellated mask in nifti format in Fiji image analysis software (v2.1.0/1.53c) using the Connected Components Labeling feature within the MorphoLibJ plugin and Nifti export plugin. A 4-D mask image from the 3-D labeled mask was generated using the PET PVC toolbox’s pvc_mask4d function.

Briefly, to prepare input data for PVC, all input PET and CT images were separately grouped and converted to a 32-bit nifti format from DICOM using the application MRIConvert (v2.1.0). The input PET nifti file was then resaved using Fiji’s Nifti plugin to ensure metadata congruence between the input data and mask.

The PVC-corrected PET images were analyzed with AMIDE (v1.0.5) and VivoQuant software (v2.5; inviCRO, LLC, Boston, MA, USA).

### Injection hold-up in large tumors

B16F10 tumors between 200–300 mm^3^ in size, were injected with 20 µL of mouse serum albumin labeled with Alexa Fluor^TM^ 647 (Invitrogen, A20006). Resected tumors were weighed and homogenized using Silica glass beads (Millipore Sigma, Z763756) with the Mini-Beadbeater-16 (Bio Spec Products Inc.) Standards were generated by mixing naive excised tumors with the injected dose ex-vivo. Each sample and standard’s fluorescence (excitation at 650 nm, emission at 690 nm, gain greater than 100, z depth at 19350 µm) was measured using an Infinite M1000 microplate reader (Tecan). Injection-hold up percentage for each sample was interpolated from a standard curve.

### Tumor immunohistochemistry

Excised tumors were fixed in 10% neutral buffered formalin overnight, embedded in paraffin and sectioned to 5 μ thickness. Prior to staining, heat-induced acid epitope retrieval was performed. Staining for collagen type I using anti-collagen type I antibody (Abcam ab34710, 1:1000) was followed by secondary staining and development using an HRP-conjugated anti-rabbit antibody (Abcam ab6721, 1:1000). Staining for EIIIB-containing fibronectin were performed with site-specifically biotinylated NJB2 was followed by secondary staining and development using an HRP-conjugated streptavidin (Abcam ab64269, 1:1000). Hematoxylin was the final counterstain. Sections were digitally scanned at ×40 magnification on a Leica Aperio AT2 Digital Pathology Slide Scanner (Leica Biosytems) and analyzed on Aperio ImageScope (v12.3.0.5056).

### Flow cytometry

Tumors were injected with proteins labeled with Alexa Fluor^TM^ 647 (Invitrogen, A20006); for each protein, the degree-of-labeling was determined to be ~1.6 dye molecules per protein. Resected tumors were weighed, dissociated into small pieces, and incubated in RPMI-1640 containing 1 mg/ml collagenase and dispase (Roche) and 25 µg/ml Dnase I (Roche) for 30 min at 37 °C. Further mechanical dissociation through a 70 µm filter was used to generate a single cell suspension for staining. Staining using antibodies to CD11b (BV 421, clone M1/70, Biolegend 101251), Ly6-C (PE-Cy7, clone HK1.4, Biolegend 128017), and F4/80 (PerCP-Cy5.5, clone BM8, Biolegend 123127), CD45 (APC-Cy7, clone 30-F11, Biolegend 103115), CD3e (BUV 395, clone 145-2C11, BD Biosciences 563565), CD8a (BUV 737, clone 53-6.7, BD Biosciences 612759), NK1.1 (PerCP-Cy5.5, clone PK136, Biolegend 108727), CD4 (PE-Cy7, clone GK1.5, Biolegend 100421), CD25 (Alexa Fluor 700, clone PC61, Biolegend 102024), and FoxP3 (PE, clone 150D, Biolegend 320007) was performed at a 1:100 dilution. Viability was assessed by LIVE/DEAD Fixable Zombie Aqua (Biolegend 423101). Fluorescence quantitation of a tumor’s total Alexa Fluor 647 content was performed using Quantum™ Alexa Fluor® 647 MESF Beads (647, Bang Labs). Data was obtained using BD FACSDiva Software v6 and analyzed using FlowJo v10.7.1.

### Reporting summary

Further information on research design is available in the Nature Research Reporting Summary linked to this article.

## Supplementary information

Supplementary Information

Reporting Summary

## Data Availability

The crystal structure of murine LAIR-1 ectodomain was obtained from PDB 4ETY [accession code]. The remaining data supporting the findings of this study are available within the Article, Supplementary Information, or Source data file. The processed data and graphs generated in MATLAB R2019b are also available on Github [accession code]. GraphPad Prism 9.0.2 was used for plotting and statistical analysis. Source data are provided with this paper.

## Source data

Source Data

## Code availability

The pharmacokinetic model, written and analyzed in MATLAB R2019b, has been published on Github. When appropriate, please cite this paper and the following code (10.5281/ZENODO.5546653).

## References

[1] Champiat S (2021). Intratumoral immunotherapy: from trial design to clinical practice. Clin. Cancer Res..

[2] Marabelle A, Tselikas L, de Baere T, Houot R (2017). Intratumoral immunotherapy: using the tumor as the remedy. Ann. Oncol..

[3] Huang A (2020). Human intratumoral therapy: linking drug properties and tumor transport of drugs in clinical trials. J. Control. Release.

[4] van der Zanden SY, Luimstra JJ, Neefjes J, Borst J, Ovaa H (2020). Opportunities for small molecules in cancer immunotherapy. Trends Immunol..

[5] Zhou S, Chen W, Cole J, Zhu G (2020). Delivery of nucleic acid therapeutics for cancer immunotherapy. Med. Drug Discov..

[6] Ray A (2016). A phase I study of intratumoral ipilimumab and interleukin-2 in patients with advanced melanoma. Oncotarget.

[7] Lundstrom K (2017). Latest trends in cancer therapy applying viral vectors. Future Virol..

[8] Research, C. M. & Case Medical Research. Safety and tolerability of SYNB1891 injection alone or in combination with atezolizumab in adult subjects. *Case Med. Res.*10.31525/ct1-nct04167137 (2019).

[9] Fröbom R (2020). Phase I trial evaluating safety and efficacy of intratumorally administered inflammatory allogeneic dendritic cells (ilixadencel) in advanced gastrointestinal stromal tumors. Cancer Immunol. Immunother..

[10] Wang Y (2017). Toward greater insights on pharmacokinetics and exposure-response relationships for therapeutic biologics in oncology drug development. Clin. Pharmacol. Ther..

[11] Jain RK (1987). Transport of molecules in the tumor interstitium: a review. Cancer Res..

[12] Rohner NA, Thomas SN (2016). Melanoma growth effects on molecular clearance from tumors and biodistribution into systemic tissues versus draining lymph nodes. J. Control. Release.

[13] Nomura T, Koreeda N, Yamashita F, Takakura Y, Hashida M (1998). Effect of particle size and charge on the disposition of lipid carriers after intratumoral injection into tissue-isolated tumors. Pharm. Res..

[14] Li Z (2019). Effect of size on solid tumor disposition of protein therapeutics. Drug Metab. Dispos..

[15] Wittrup KD, Thurber GM, Schmidt MM, Rhoden JJ (2012). Practical theoretic guidance for the design of tumor-targeting agents. Methods Enzymol..

[16] Schmidt MM, Wittrup KD (2009). A modeling analysis of the effects of molecular size and binding affinity on tumor targeting. Mol. Cancer Ther..

[17] Orcutt KD, Rhoden JJ, Ruiz-Yi B, Frangioni JV, Dane Wittrup K (2012). Effect of small-molecule–binding affinity on tumor uptake in vivo: a systematic study using a pretargeted bispecific antibody. Mol. Cancer Ther..

[18] Thurber GM, Schmidt MM, Wittrup KD (2008). Factors determining antibody distribution in tumors. Trends Pharmacol. Sci..

[19] Thurber GM, Schmidt MM, Wittrup KD (2008). Antibody tumor penetration: transport opposed by systemic and antigen-mediated clearance. Adv. Drug Deliv. Rev..

[20] Thurber GM, Zajic SC, Wittrup KD (2007). Theoretic criteria for antibody penetration into solid tumors and micrometastases. J. Nucl. Med..

[21] Li Z, Li Y, Chang HP, Yu X, Shah DK (2021). Two-pore physiologically based pharmacokinetic model validation using whole-body biodistribution of trastuzumab and different-size fragments in mice. J. Pharmacokinet. Pharmacodyn..

[22] Den Otter W (2008). Local therapy of cancer with free IL-2. Cancer Immunol. Immunother..

[23] Langan EA (2019). Intralesional interleukin-2: a novel option to maximize response to systemic immune checkpoint therapy in loco-regional metastatic melanoma. Dermatol. Ther..

[24] Mattijssen V (1994). Intratumoral PEG-interleukin-2 therapy in patients with locoregionally recurrent head and neck squamous-cell carcinoma. Ann. Oncol..

[25] Neri D (2014). Intralesional treatment of stage III metastatic melanoma patients with L19-IL2: clinical and systemic immunological responses. J. Clin. Orthod..

[26] Danielli R (2015). Intralesional administration of L19-IL2/L19-TNF in stage III or stage IVM1a melanoma patients: results of a phase II study. Cancer Immunol. Immunother..

[27] Albertini MR (2012). Phase II trial of hu14.18-IL2 for patients with metastatic melanoma. Cancer Immunol. Immunother..

[28] Connor JP (2013). A phase 1b study of humanized KS-interleukin-2 (huKS-IL2) immunocytokine with cyclophosphamide in patients with EpCAM-positive advanced solid tumors. BMC Cancer.

[29] Momin, N. et al. Anchoring of intratumorally administered cytokines to collagen safely potentiates systemic cancer immunotherapy. *Sci. Transl. Med*. **11**, eaaw2614 (2019). 10.1126/scitranslmed.aaw2614 PMC7811803 31243150

[30] Alexandrakis G (2004). Two-photon fluorescence correlation microscopy reveals the two-phase nature of transport in tumors. Nat. Med..

[31] Li Y (2020). Multifunctional oncolytic nanoparticles deliver self-replicating IL-12 RNA to eliminate established tumors and prime systemic immunity. Nat. Cancer.

[32] Ishihara, J. et al. Matrix-binding checkpoint immunotherapies enhance antitumor efficacy and reduce adverse events. *Sci. Transl. Med*. **9**, eaan0401 (2017). 10.1126/scitranslmed.aan0401 29118259

[33] Ishihara J (2018). Improving efficacy and safety of agonistic anti-CD40 antibody through extracellular matrix affinity. Mol. Cancer Ther..

[34] Ishihara, J. et al. Targeted antibody and cytokine cancer immunotherapies through collagen affinity. *Sci. Transl. Med*. **11**, eaau3259 (2019). 10.1126/scitranslmed.aau3259 PMC6541444 30971453

[35] Lebbink RJ (2006). Collagens are functional, high affinity ligands for the inhibitory immune receptor LAIR-1. J. Exp. Med..

[36] Chao G (2006). Isolating and engineering human antibodies using yeast surface display. Nat. Protoc..

[37] Farndale RW (2008). Cell–collagen interactions: the use of peptide Toolkits to investigate collagen–receptor interactions. Biochem. Soc. Trans..

[38] Brondijk THC (2010). Crystal structure and collagen-binding site of immune inhibitory receptor LAIR-1: unexpected implications for collagen binding by platelet receptor GPVI. Blood.

[39] Andersen JT (2012). Structure-based mutagenesis reveals the albumin-binding site of the neonatal Fc receptor. Nat. Commun..

[40] Zhu EF (2015). Synergistic innate and adaptive immune response to combination immunotherapy with anti-tumor antigen antibodies and extended serum half-life IL-2. Cancer Cell.

[41] Kwan BH (2017). Integrin-targeted cancer immunotherapy elicits protective adaptive immune responses. J. Exp. Med..

[42] Dréau D (2019). Combining the specific anti-MUC1 antibody TAB004 and lip-MSA-IL-2 limits pancreatic cancer progression in immune competent murine models of pancreatic ductal adenocarcinoma. Front. Oncol..

[43] Sun Z (2019). A next-generation tumor-targeting IL-2 preferentially promotes tumor-infiltrating CD8+ T-cell response and effective tumor control. Nat. Commun..

[44] Muñoz, N. M. et al. Influence of injection technique, drug formulation and tumor microenvironment on intratumoral immunotherapy delivery and efficacy. *J. Immunother. Cancer* **9**, e001800 (2021). 10.1136/jitc-2020-001800 PMC7887346 33589526

[45] Wang Y (2003). Systemic dissemination of viral vectors during intratumoral injection. Mol. Cancer Ther..

[46] Stylianopoulos T, Munn LL, Jain RK (2018). Reengineering the physical microenvironment of tumors to improve drug delivery and efficacy: From mathematical modeling to bench to bedside. Trends Cancer.

[47] Padera TP (2002). Lymphatic metastasis in the absence of functional intratumor lymphatics. Science.

[48] Hagendoorn J (2006). Onset of abnormal blood and lymphatic vessel function and interstitial hypertension in early stages of carcinogenesis. Cancer Res..

[49] Momin, N. *noormomin/Intratumoral-Immunotherapy-PK: v1.0*. (Zenodo, 2021). 10.5281/ZENODO.5546653.

[50] Charych D (2017). Modeling the receptor pharmacology, pharmacokinetics, and pharmacodynamics of NKTR-214, a kinetically-controlled interleukin-2 (IL2) receptor agonist for cancer immunotherapy. PLoS ONE.

[51] Lechner MG (2013). Immunogenicity of murine solid tumor models as a defining feature of in vivo behavior and response to immunotherapy. J. Immunother..

[52] Riegler J (2018). Tumor elastography and its association with collagen and the tumor microenvironment. Clin. Cancer Res..

[53] Krol A, Maresca J, Dewhirst MW, Yuan F (1999). Available volume fraction of macromolecules in the extravascular space of a fibrosarcoma: implications for drug delivery. Cancer Res..

[54] Thurber GM, Wittrup KD (2008). Quantitative spatiotemporal analysis of antibody fragment diffusion and endocytic consumption in tumor spheroids. Cancer Res..

[55] Madsen DH (2017). Tumor-associated macrophages derived from circulating inflammatory monocytes degrade collagen through cellular uptake. Cell Rep..

[56] Netti PA, Berk DA, Swartz MA, Grodzinsky AJ, Jain RK (2000). Role of extracellular matrix assembly in interstitial transport in solid tumors. Cancer Res..

[57] Schwarzbauer JE, Patel RS, Fonda D, Hynes RO (1987). Multiple sites of alternative splicing of the rat fibronectin gene transcript. EMBO J..

[58] Jailkhani N (2019). Noninvasive imaging of tumor progression, metastasis, and fibrosis using a nanobody targeting the extracellular matrix. Proc. Natl Acad. Sci. USA.

[59] Centanni M, Moes DJAR, Trocóniz IF, Ciccolini J, van Hasselt JGC (2019). Clinical pharmacokinetics and pharmacodynamics of immune checkpoint inhibitors. Clin. Pharmacokinet..

[60] Feng Y (2014). Model-based clinical pharmacology profiling of ipilimumab in patients with advanced melanoma. Br. J. Clin. Pharmacol..

[61] Ziffels B, Pretto F, Neri D (2018). Intratumoral administration of IL2- and TNF-based fusion proteins cures cancer without establishing protective immunity. Immunotherapy.

[62] Naba A (2012). The matrisome: in silico definition and in vivo characterization by proteomics of normal and tumor extracellular matrices. Mol. Cell. Proteom..

[63] Francis, D. M. et al. Blockade of immune checkpoints in lymph nodes through locoregional delivery augments cancer immunotherapy. *Sci. Transl. Med*. **12**, eaay3575 (2020). 10.1126/scitranslmed.aay3575 PMC8377700 32998971

[64] Mehta NK (2020). Pharmacokinetic tuning of protein–antigen fusions enhances the immunogenicity of T-cell vaccines. Nat. Biomed. Eng..

[65] Jain RK, Tong RT, Munn LL (2007). Effect of vascular normalization by antiangiogenic therapy on interstitial hypertension, peritumor edema, and lymphatic metastasis: insights from a mathematical model. Cancer Res..

[66] Li Z (2021). A two-pore physiologically based pharmacokinetic model to predict subcutaneously administered different-size antibody/antibody fragments. AAPS J..

[67] Michallet M (2004). Pegylated recombinant interferon alpha-2b vs recombinant interferon alpha-2b for the initial treatment of chronic-phase chronic myelogenous leukemia: a phase III study. Leukemia.

[68] Jacquelot N (2019). Sustained Type I interferon signaling as a mechanism of resistance to PD-1 blockade. Cell Res..

[69] Thibaut R (2020). Bystander IFN-γ activity promotes widespread and sustained cytokine signaling altering the tumor microenvironment. Nat. Cancer.

[70] Garcia-Diaz A (2019). Interferon receptor signaling pathways regulating PD-L1 and PD-L2 expression. Cell Rep..

[71] Pai C-CS (2019). Clonal deletion of tumor-specific T cells by interferon-γ confers therapeutic resistance to combination immune checkpoint blockade. Immunity.

[72] Boucher Y, Brekken C, Netti PA, Baxter LT, Jain RK (1998). Intratumoral infusion of fluid: estimation of hydraulic conductivity and implications for the delivery of therapeutic agents. Br. J. Cancer.

[73] Sharma J, Lv H, Gallo JM (2013). Intratumoral modeling of gefitinib pharmacokinetics and pharmacodynamics in an orthotopic mouse model of glioblastoma. Cancer Res..

[74] Singh AP (2020). Evolution of the systems pharmacokinetics-pharmacodynamics model for antibody-drug conjugates to characterize tumor heterogeneity and in vivo bystander effect. J. Pharmacol. Exp. Ther..

[75] Khera E (2021). Quantifying ADC bystander payload penetration with cellular resolution using pharmacodynamic mapping. Neoplasia.

[76] Li H-L (2008). Pharmacokinetic and pharmacodynamic study of intratumoral injection of an adenovirus encoding endostatin in patients with advanced tumors. Gene Ther..

[77] Sheth RA (2020). Assessment of image-guided intratumoral delivery of immunotherapeutics in patients with cancer. JAMA Netw. Open.

[78] FDA. *US Food and drug administration. Package Insert - IMLYGIC*. https://www.fda.gov/media/94129/.

[79] Van Deventer JA, Wittrup KD (2014). Yeast surface display for antibody isolation: library construction, library screening, and affinity maturation. Methods Mol. Biol..

[80] Angelini A (2015). Protein engineering and selection using yeast surface display. Methods Mol. Biol..

[81] Knight CG, Onley CM, Farndale RW (2004). Peptide synthesis in the study of collagen-platelet interactions. Methods Mol. Biol..

[82] Thomas BA (2016). PETPVC: a toolbox for performing partial volume correction techniques in positron emission tomography. Phys. Med. Biol..

[83] Erlandsson K, Buvat I, Pretorius PH, Thomas BA, Hutton BF (2012). A review of partial volume correction techniques for emission tomography and their applications in neurology, cardiology and oncology. Phys. Med. Biol..

