# Supplementary material for: Maximizing response to intratumoral immunotherapy in mice by tuning local retention
Source: Nat Commun. 2022 Jan 10;13:109. doi: 10.1038/s41467-021-27390-6 (PMC8748612; doi:10.1038/s41467-021-27390-6)
Supplement: Supplementary file 1 — Supplementary Information [file 41467_2021_27390_MOESM1_ESM.pdf]

Inventory of Supplementary Information

Supplementary Figure 1. Collagen-related peptide (CRP) binds yeast-surface displayed LAIR.

Supplementary Figure 2. Sorted yeast accumulate mutations that weaken LAIR's binding to CRP.

Supplementary Figure 3. Characterization of sorted LAIR clones on-yeast with CRP differs off-yeast with native collagen.

Supplementary Figure 4. Recombinant LAIR- and LAIRx- IL-2 fusion proteins are stable and monomeric.

Supplementary Figure 5. Mutations that lend higher affinity CRP binding on yeast do not translate to higher affinity native collagen binding.

Supplementary Figure 6: Immune-infiltration status of established B16F10 tumors

Supplementary Figure 7. LAIR-IL2 and LAIRx-LAIRx-MSA^{H464Q}-IL2 are therapeutically identical in a setting of matched systemic exposure

Supplementary Figure 8. Intratumoral amount and fractional activity varies by molecular weight and collagen affinity of injected agonists

Supplementary Figure 9. Model predict blood clearance eliminates most intratumorally-injected IL-2 fusion protein

Supplementary Figure 10. Model output is sensitive to inputted injected-dose, tumor collagen density, and collagen turnover

Supplementary Figure 11. Model inputs, collagen concentration and turnover, empirically measured and validated

Supplementary Figure 12. Characterization of fusion proteins tracked by PET imaging

Supplementary Figure 13. Intratumorally-injected proteins differentially escape tumor overtime

Supplementary Figure 14. Injected volume exceeding B16F10 hold-up volume leads to systemic drug dissemination.

Supplementary Figure 15. Modeled rate of tumor escape of injected proteins agrees with PET measured rate of escape

Supplementary Figure 16. Protein quantification from PET image validated by gamma counter measurement

Supplementary Figure 17 Maximum intensity projection reveals peritumoral accumulation of intratumorally injected proteins

Supplementary Figure 18 Recombinant NJB2- and NJT6- IL-2 fusion proteins are monomeric and bioactive.

Supplementary Table 1: Glossary of compartment model variables and inputs

Supplementary Table 2. Initial conditions inputted in compartment model

Supplementary Table 3. Ordinary differential equations in the compartment model

Supplementary Table 4. Amino acid sequence of all novel fusion proteins

Supplementary References

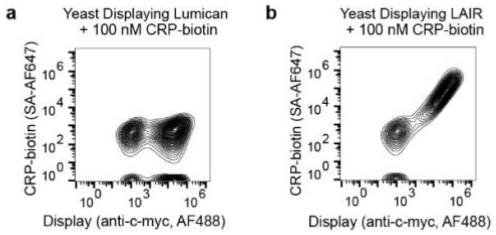

Supplementary Fig 1

Collagen-related peptide (CRP) binds yeast-surface displayed LAIR.

Flow cytometry plots of yeast displaying the protein lumican, unable to bind biotinylated CRP; and **b**, LAIR able to specifically bind biotinylated collagen related peptide (CRP). Binding to CRP was detected by fluorescently labeled secondary antibodies to c-myc and fluorescently labeled streptavidin. SA-AF647, Alexa Fluor 647 labeled streptavidin; AF488, Alexa Fluor 488.

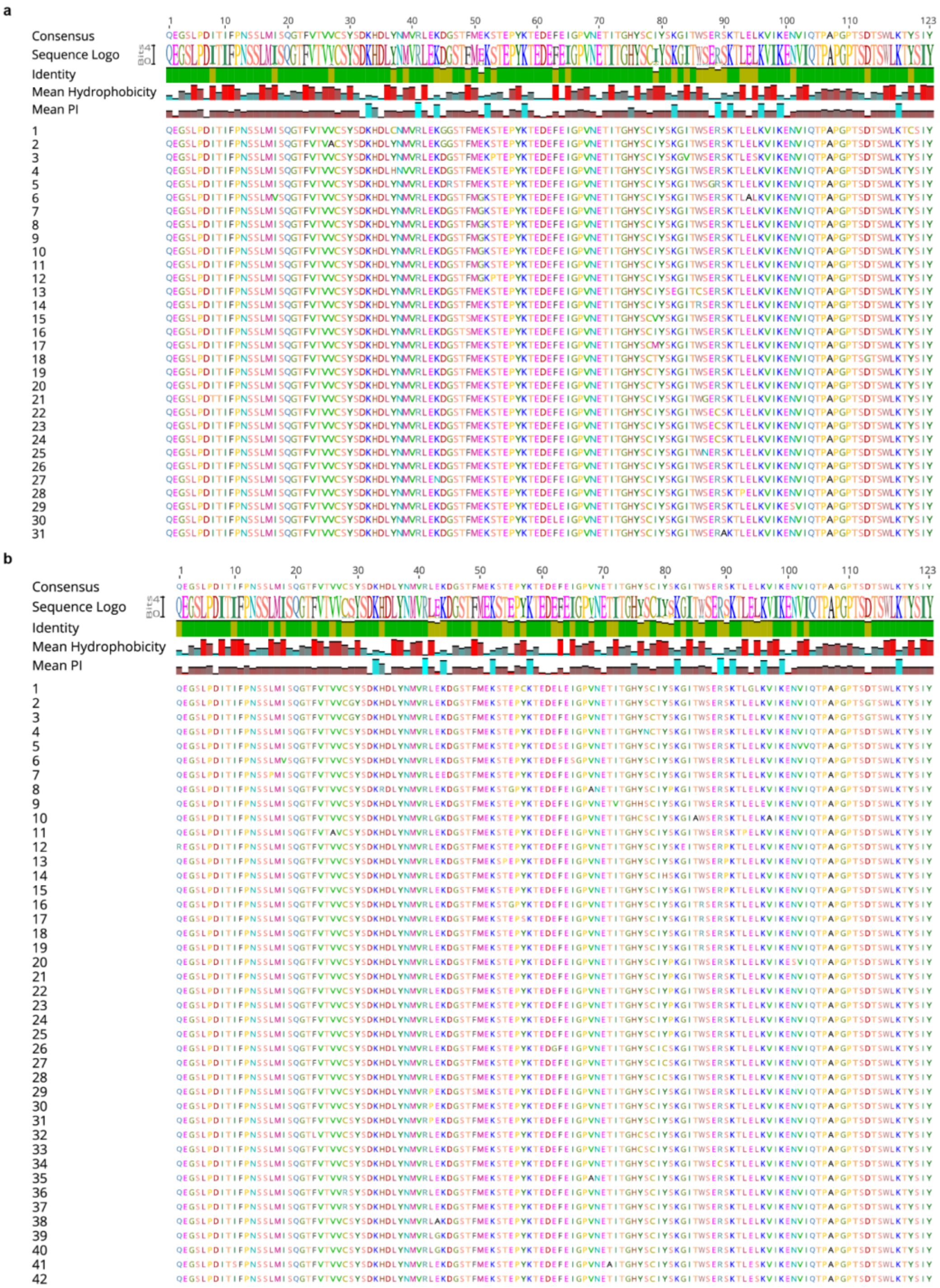

Supplementary Fig 2

Sorted yeast accumulate mutations that weaken LAIR's binding to CRP.

a, Sequences of enriched yeast clones after sorting the original mutagenized LAIR library displayed in Fig. 1b (left panel) and **b,** after sorting the isolated clones displayed in Fig. 1b (right panel).

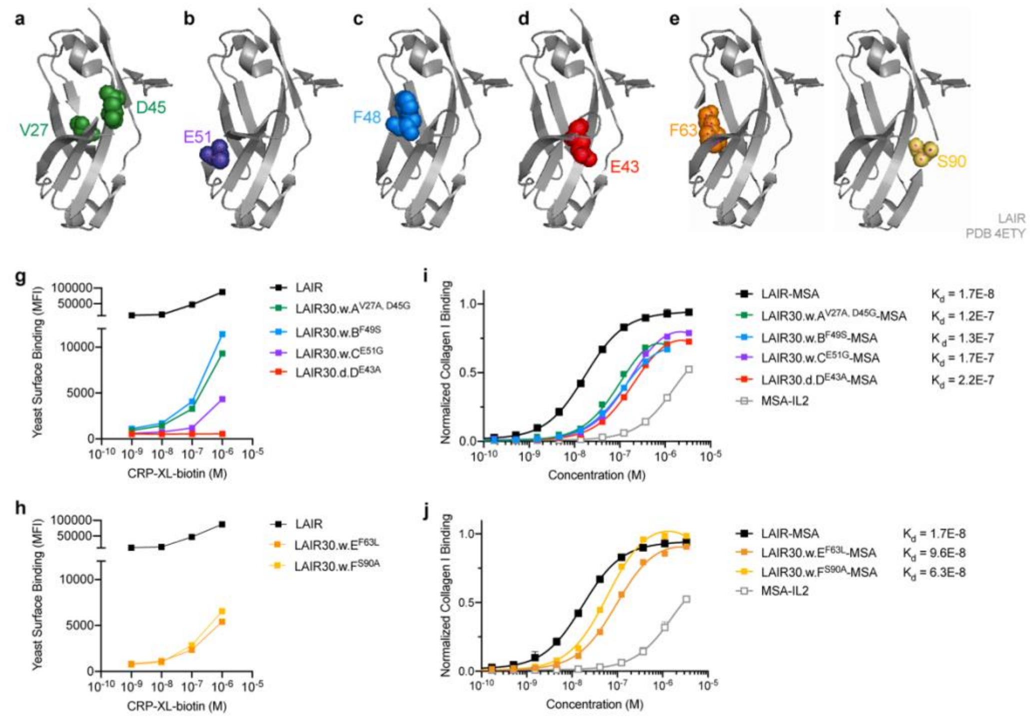

Supplementary Fig 3

Characterization of sorted LAIR clones on-yeast with CRP differs from off- yeast with native collagen.

Ribbon representation of LAIR (PDB 4ETY: http://doi.org/10.2210/pdb4ety/pdb) with colored residues demarking mutations in **a**, LAIR30.w.A^{V27A,D45G} **b**, LAIR30.w.B^{F49S} **c**, LAIR30.w.C^{E51G} **d**, LAIR30.d.D^{E43A} **e**, LAIR30.w.E^{F63L} **f**, LAIR30.w.F^{S90A}. **g**, Binding titration of biotinylated CRP with mutants in panels **a-d** compared to wild-type LAIR-displaying yeast. Yeast surface binding was measured by median fluorescence intensity (MFI) of streptavidin-Alexa Fluor 647 by flow cytometry (mean, n = 1) **h**, Binding titration of biotinylated CRP with mutants in panels **e-f** compared to wild-type LAIR-displaying yeast. Yeast surface binding was measured by median fluorescence intensity (MFI) of streptavidin-Alexa Fluor 647 by flow cytometry (mean, n = 1). **i,** Binding to collagen type I of mutants in panels **a-d** as fusion proteins to mouse serum albumin (MSA) measured by enzyme-linked immunosorbent assay (ELISA) (mean; n = 2;). **j,** Binding to collagen type I of enriched mutants from panels **e-f** as fusion proteins to mouse serum albumin (MSA) measured by ELISA (mean; n = 2).

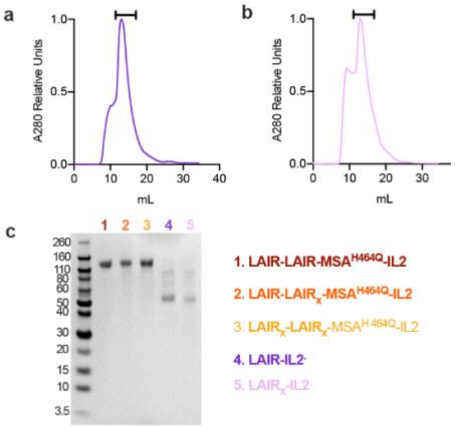

Supplementary Fig 4

Recombinant LAIR- and LAIR$_x$- IL-2 fusion proteins are stable and monomeric.

Representative absorbance spectrum of **a,** LAIR-IL2 and **b,** LAIR$_x$-IL2 (bracketed) purified by size-exclusion chromatography. **C**, Purified IL-2 fusion proteins visualized by SDS-PAGE with Coomassie blue under non-reducing conditions. This experiment was repeated three times with identical results.

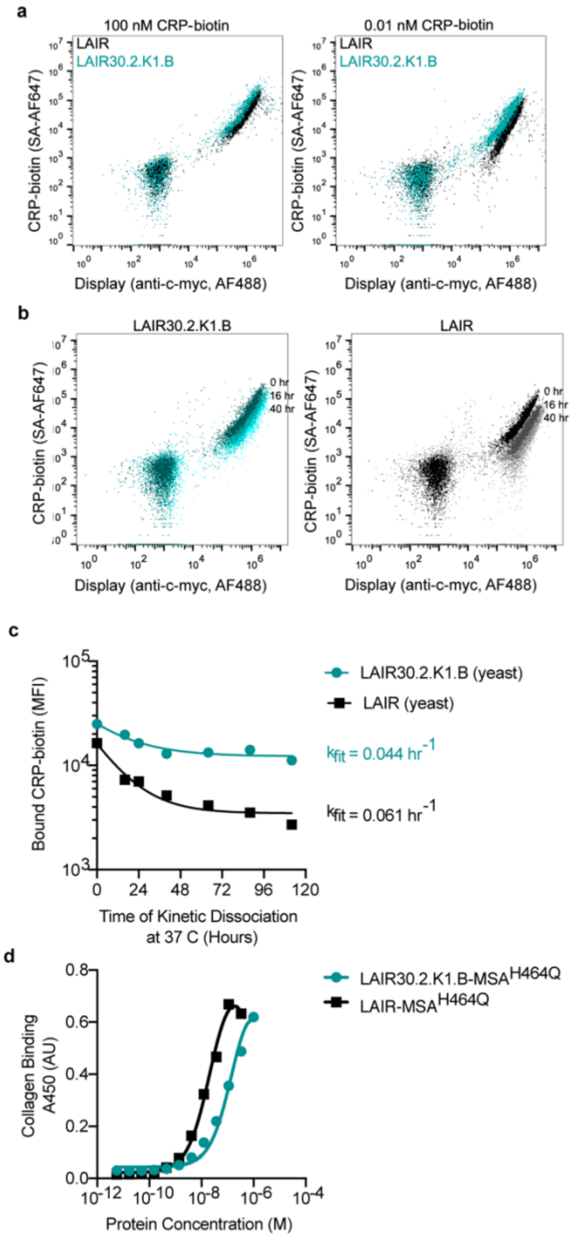

Supplementary Fig 5

Mutations that lend higher affinity CRP binding on yeast do not translate to higher affinity native collagen binding.
a, Flow cytometry plots of yeast displaying clone LAIR30.2.K1.B or wild-type LAIR after equilibration with 100 nM (left) or 0.01 nM (right) of biotinylated collagen-related peptide (CRP). **b,** Flow cytometry plots of residual biotinylated CRP binding after saturated yeast displaying LAIR30.2.K1.B (left) or wild-type LAIR (right) underwent kinetic dissociation and competition with 100 nM of non-biotinylated CRP for 0, 16 and 24 hours. **c,** Quantification of residual CRP-biotin binding during kinetic dissociation and competition (including panel **b**). Binding was measured by median fluorescence intensity (MFI) of streptavidin-Alexa Fluor 647. The overall rate of CRP-biotin unbinding (k_{fit}) was determined by fitting a one phase exponential decay. SA-AF647, Alexa Fluor 647 labeled streptavidin; AF488, Alexa Fluor 488. **C,** Binding to collagen type I of LAIR30.2.K1.B and wild-type LAIR fused to mouse serum albumin bearing histidine to glutamic acid mutation (MSA^{H464Q}) was measured by enzyme-linked immunosorbent assay (ELISA) (*n* = 1).

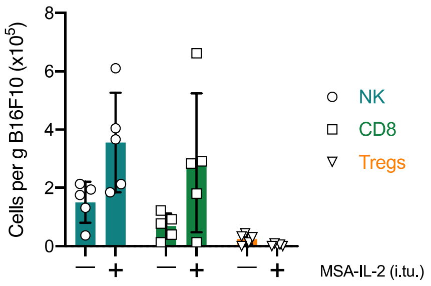

Supplementary Fig 6

Immune-infiltration status of established B16F10 tumors

Mice were inoculated with 1x10^6 B16F10 cells subcutaneously in the right flank on day 0. B16F10 tumors were excised four days after treatment with 100 ug of TA99 intraperitoneally and intratumoral injection of either PBS or MSA-IL2 on day 6. Excised tumors were analyzed by flow cytometry for infiltration of NK cells, CD8+ T cells, and Tregs (mean +/- s.d., *n* = 5).

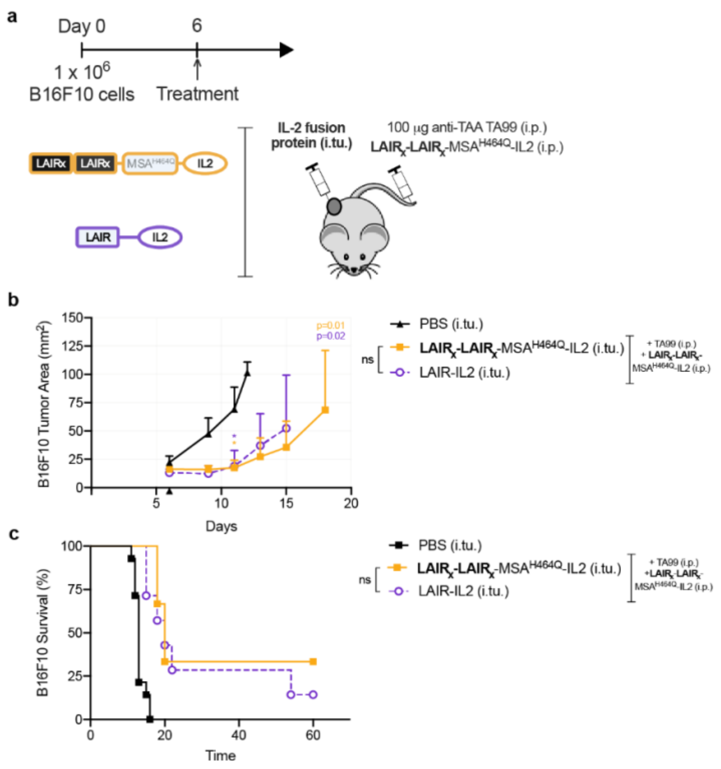

Supplementary Fig 7

LAIR-IL2 and LAIR$_x$-LAIR$_x$-MSA^{H464Q}-IL2 are therapeutically identical in a setting of matched systemic exposure

a, Schematic of the B16F10 tumour immunotherapy study timeline (top) and treatment components (bottom). For IL-2 fusion proteins, the lines connecting LAIR, LAIR$_x$, MSA^{H464Q}, and IL-2 protein domains represent glycine-serine linkers. Mice were inoculated with 1x10^6 B16F10 cells subcutaneously in the right flank on day 0. Six days after tumor implantation, treatments were initiated following the timeline. TAA, tumor associated antigen; i.tu., intratumoral; i.p., intraperitoneal. **b,** Tumor growth over time (mean + s.d.) and **c,** survival of the indicated groups (n = 14 mice for the PBS (i.tu.) group, and n = 6 mice for all other groups). Tumor area for each group is shown until a mouse in the group is euthanized. Statistical significance of each treated group's tumor area (displayed on top right corner) was calculated by using a one-tailed Student's t-test versus the PBS (i.tu.) group on day 11. Survival comparisons (displayed by the legend) were generated by a log-rank Mantel-Cox test. n.s., not significant. Panel a created with BioRender.com.

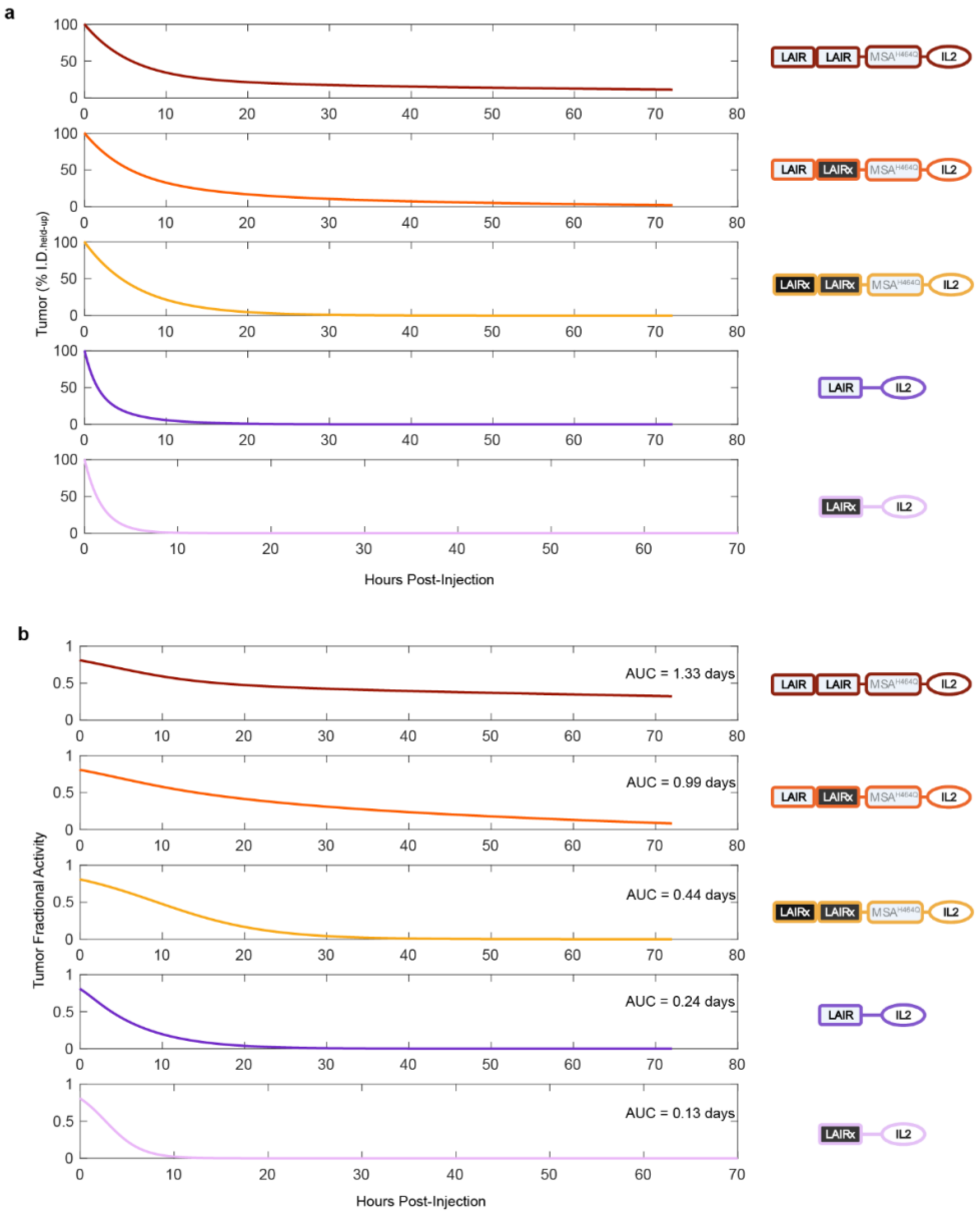

Supplementary Fig 8

Intratumoral amount and fractional activity varies by molecular weight and collagen affinity of injected agonists

Model simulation of **a**, the amount and **b**, the fractional activity of IL-2 fusion proteins in the tumor over time after *in silico* intratumoral injection for LAIR-LAIR-MSA^{H464Q}-IL2, LAIR-LAIR$_x$-MSA^{H464Q}-IL2, LAIR$_x$-LAIR$_x$-MSA^{H464Q}-IL2, LAIR-IL2, and LAIR$_x$-IL2. I.D.$_{held-up}$, injected dose held-up in the tumor immediately after intratumoral injection; AUC, area-under-curve.

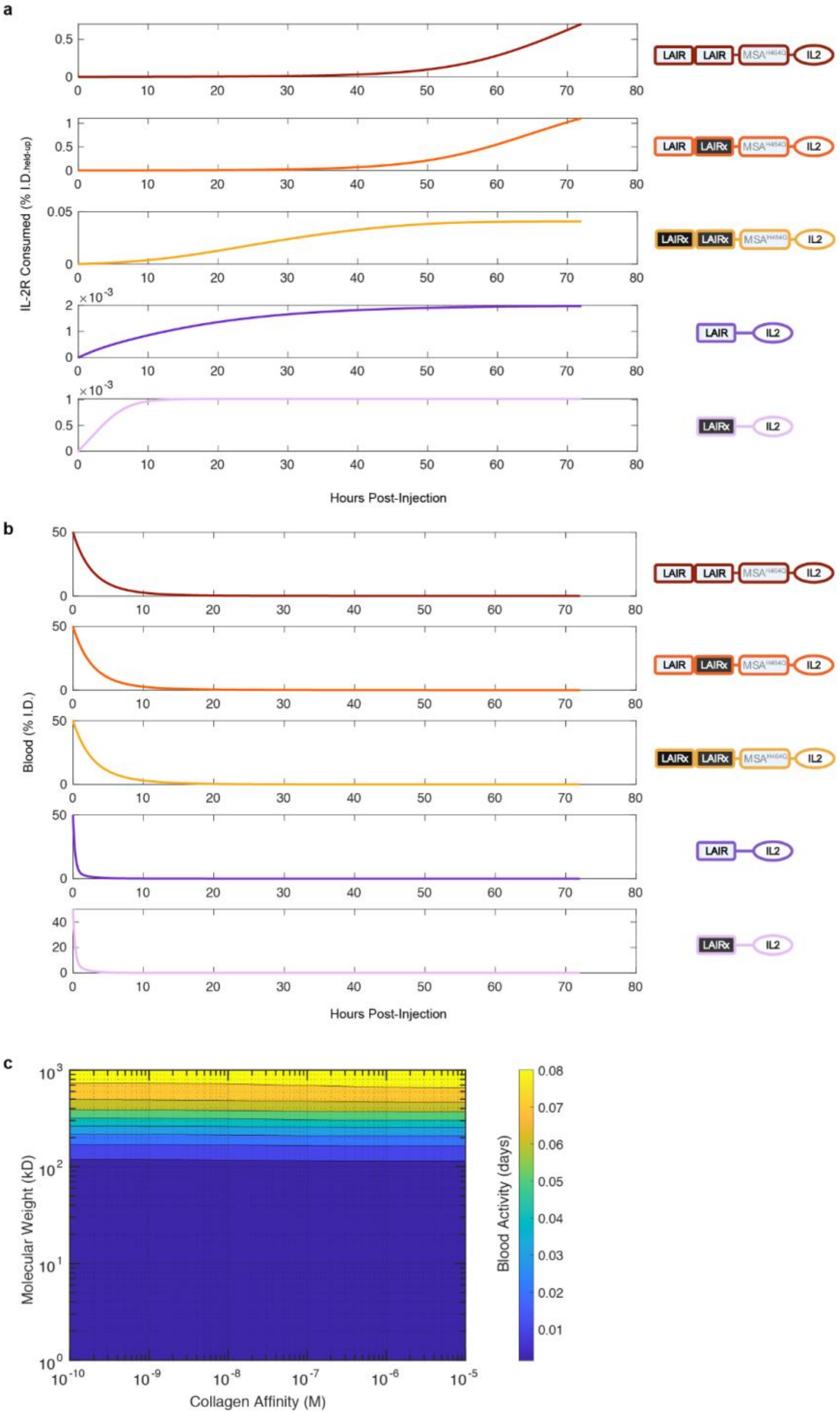

Supplementary Fig 9

Model predict blood clearance eliminates most intratumorally-injected IL-2 fusion protein

Model simulation of **a,** the cumulative total of IL-2 fusion protein internalized by IL-2R+ cells in the tumor and **b**, the amount of IL-2 fusion protein in the blood over time after *in silico* intratumoral injection for LAIR-LAIR-MSA^{H464Q}-IL2, LAIR-LAIR$_x$-MSA^{H464Q}-IL2, LAIR$_x$-LAIR$_x$-MSA^{H464Q}-IL2, LAIR-IL2, and LAIR$_x$-IL2. I.D.$_{held-up}$, injected dose held-up in the tumor immediately after intratumoral injection; I.D, total intratumorally injected dose. **c**, Model prediction of the blood activity duration (in days) of an injected IL-2 fusion protein varying in molecular weight (kD) and collagen affinity (K$_d$, in M units).

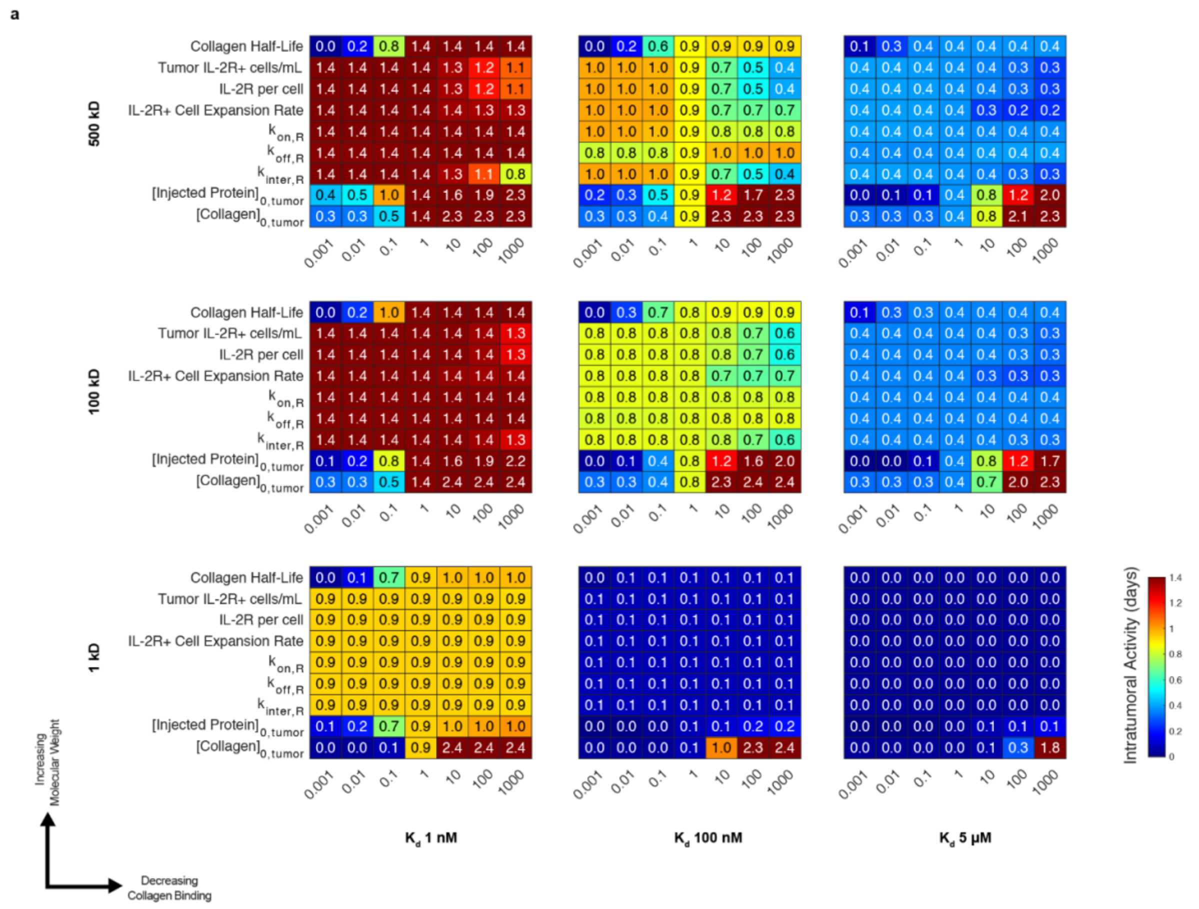

Supplementary Fig 10

Model output is sensitive to inputted injected-dose, tumor collagen density, and collagen turnover

Duration of intratumoral activity for nine theoretical proteins of different collagen affinity (column, left to right: 1 nM, 100 nM, 5 uM) and molecular weight (rows, top to bottom: 500 kDa, 100 kDa, 1 kDa) each simulated with inputs (inset rows) varying several orders of magnitude from their baseline value (inset columns: 0.001, 0.01, 0.1, 1, 10, 100, 1000). The inputs varied (and their baseline values) were: the half-life of collagen (30 days), intratumoral IL-2 receptor cell density (150E3 cells/mL), IL-2 receptors per cell (1000), IL-2R+ cell expansion rate (1 day^{-1}), $k_{on,IL2}$ (1.26E6 $M^{-1}s^{-1}$), $k_{off,IL2}$ (0.301 M^{-1}), $k_{inter,R}$ (0.4 hr^{-1}), initial intratumoral concentration of injected protein ([Injected Protein]$_{0,Tumor}$ 1E-6 M) and initial intratumoral collagen concentration ([Collagen]$_{0,Tumor}$ 2E-7 M).

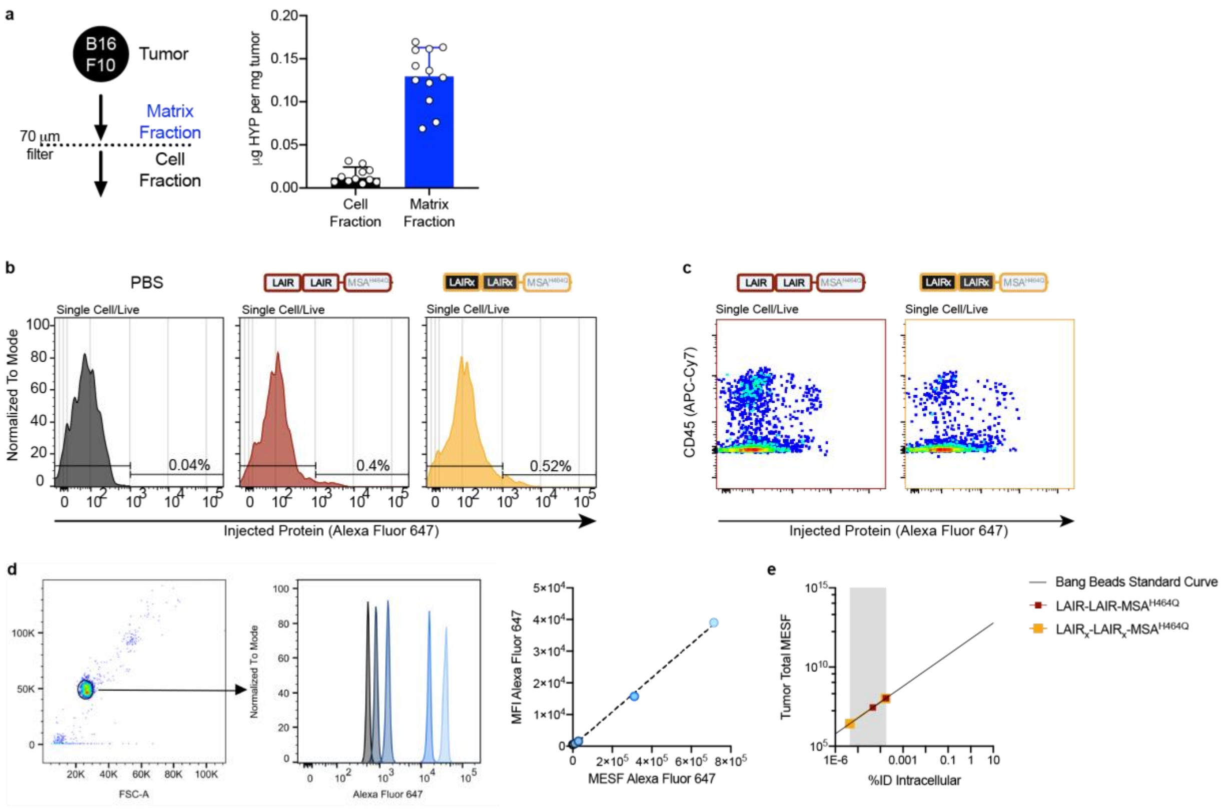

Supplementary Fig 11

Model inputs, collagen concentration and turnover, empirically measured and validated

a, Schematic depicting the separation of cellular and matrix fractions from excised B16F10 tumors (left). Quantification (right) of hydroxyproline content in the cellular (mean + s.d., *n* =11) and matrix (mean + s.d., *n* =12) fractions of a B16F10 tumor. **b**, Tumors excised 24 hours after injection with PBS (left), Alexa Fluor 647-labeled LAIR-LAIR-MSA^{H464Q} (middle), and labeled LAIR$_x$-LAIR$_x$-MSA^{H464Q} (right) analyzed by flow cytometry for cellular uptake of protein. **c**, Cells in panel b analyzed for cellular uptake of protein and expression of immune lineage marker CD45. **d**, Flow cytometry Alexa Fluor 647 median fluorescence intensity (MFI) converted into its molecules-of-equivalent-soluble-fluorochrome (MESF) based on calibration curve (right) established with fluorescence calibration beads (left, middle). **e**, Total intracellular MESF recovered from tumors analyzed in panel b (*n* = 4) compared to theoretical total tumor MESF based on the intratumorally injected protein's dose and degree of labeling.

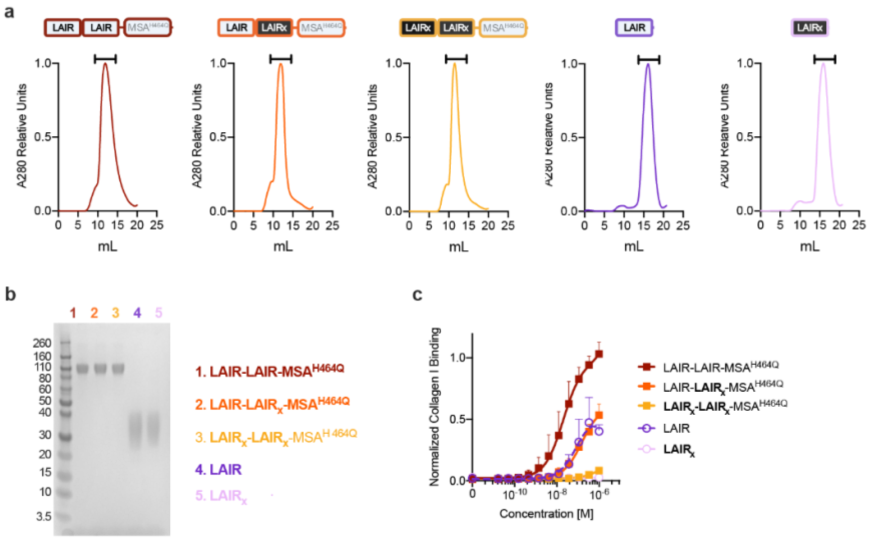

Supplementary Fig 12

Characterization of fusion proteins tracked by PET imaging

Representative absorbance spectrum of **a,** (left to right) LAIR-LAIR-MSA^{H464Q}, LAIR-LAIR$_x$-MSA^{H464Q}, LAIR$_x$-LAIR$_x$-MSA^{H464Q}, LAIR, and LAIR$_x$ purified (bracketed) by size-exclusion chromatography. **b**, Purified fusion proteins visualized by SDS-PAGE with Coomassie blue under non-reducing conditions. This experiment was repeated three times with identical results. **c**, Collagen binding of desferal labeled-IL-2 fusion proteins to collagen type I measured by ELISA (mean + s.d; *n* = 6 for all groups except *n* = 3 for LAIR$_x$)

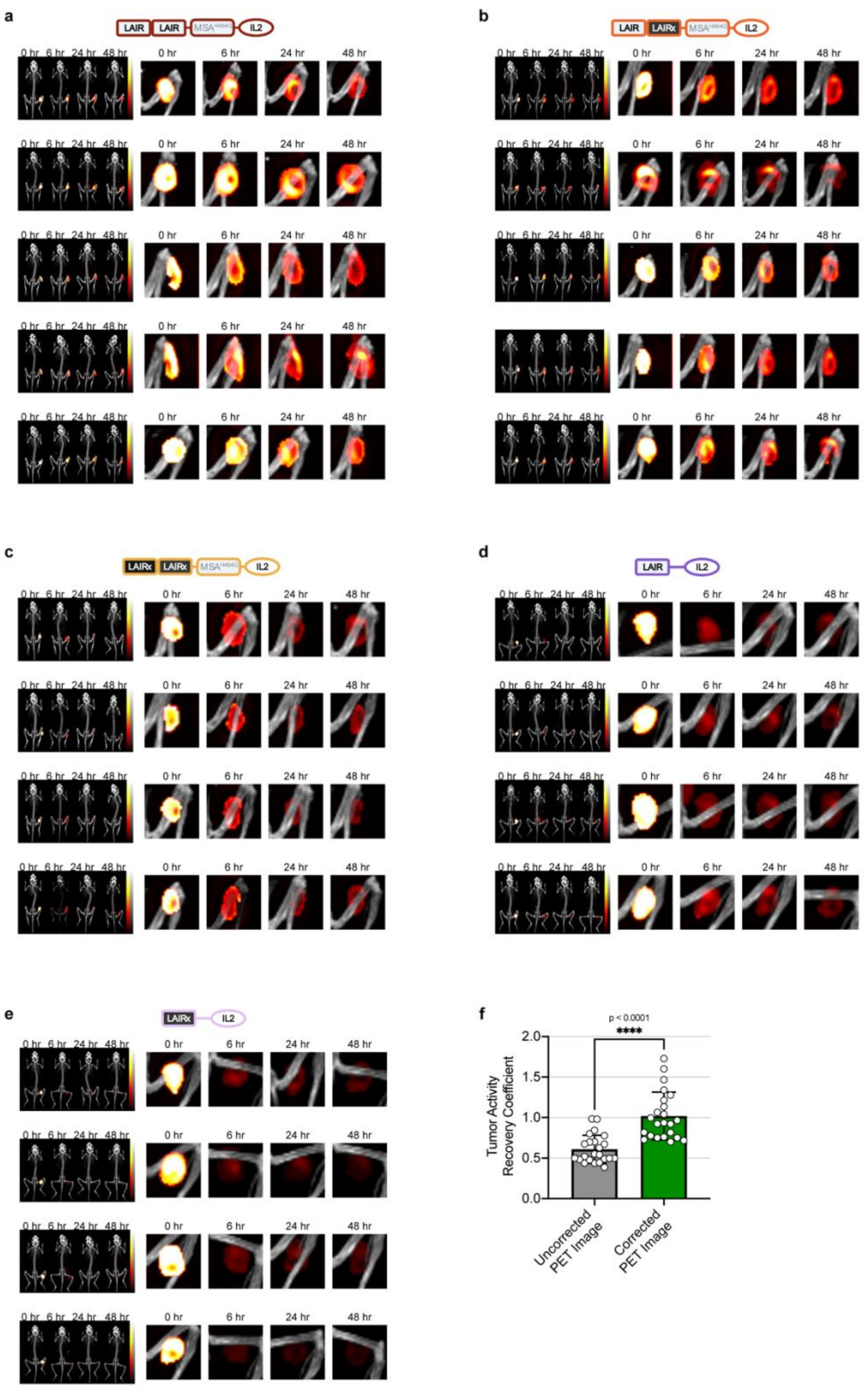

Supplementary Fig 13

Intratumorally-injected proteins differentially escape tumor overtime

Isotope decay- and PVC-corrected whole body PET/CT 3D intensity projections of B16F10 tumor-bearing mice at 0, 6, 24 and 48 hours after intratumoral injection of ^{89}Zr-labeled fusion proteins **a**, LAIR-LAIR-MSA^{H464Q} (*n* = 5) **b**, LAIR-LAIR$_x$-MSA^{H464Q} (*n* = 5); **c**, LAIR$_x$-LAIR$_x$-

MSA^{H464Q} ($n = 4$); **d**, LAIR ($n = 4$); and **e**, $LAIR_x$ ($n = 4$) **a-e**, Injected-B16F10 tumors in whole body PET/CT 3D projections (left) are enlarged (right) to visualize injected proteins. CT image (colorbar in grayscale, Hounsfield units) is used for anatomical reference. PET emission (color bar in red-yellow, from 0 – 100 %ID) is normalized for all images to the maximum activity intensity at time 0 to allow inter- and intra-group comparisons. **F**, Recovery coefficient for tumors in uncorrected PET images (mean + s.d.; $n = 23$) and partial-volume corrected PET images (mean + s.d.; $n = 23$). The recovery coefficient is defined as the ratio of activity within the segmented region of a PET image compared to the ground truth. Ground truth is established by gamma-counter quantification of ^{89}Zr in excised tumors immediately after PET imaging. Statistical significance was calculated by using a one-tailed Student's t-test. ****, $P < 0.0001$.

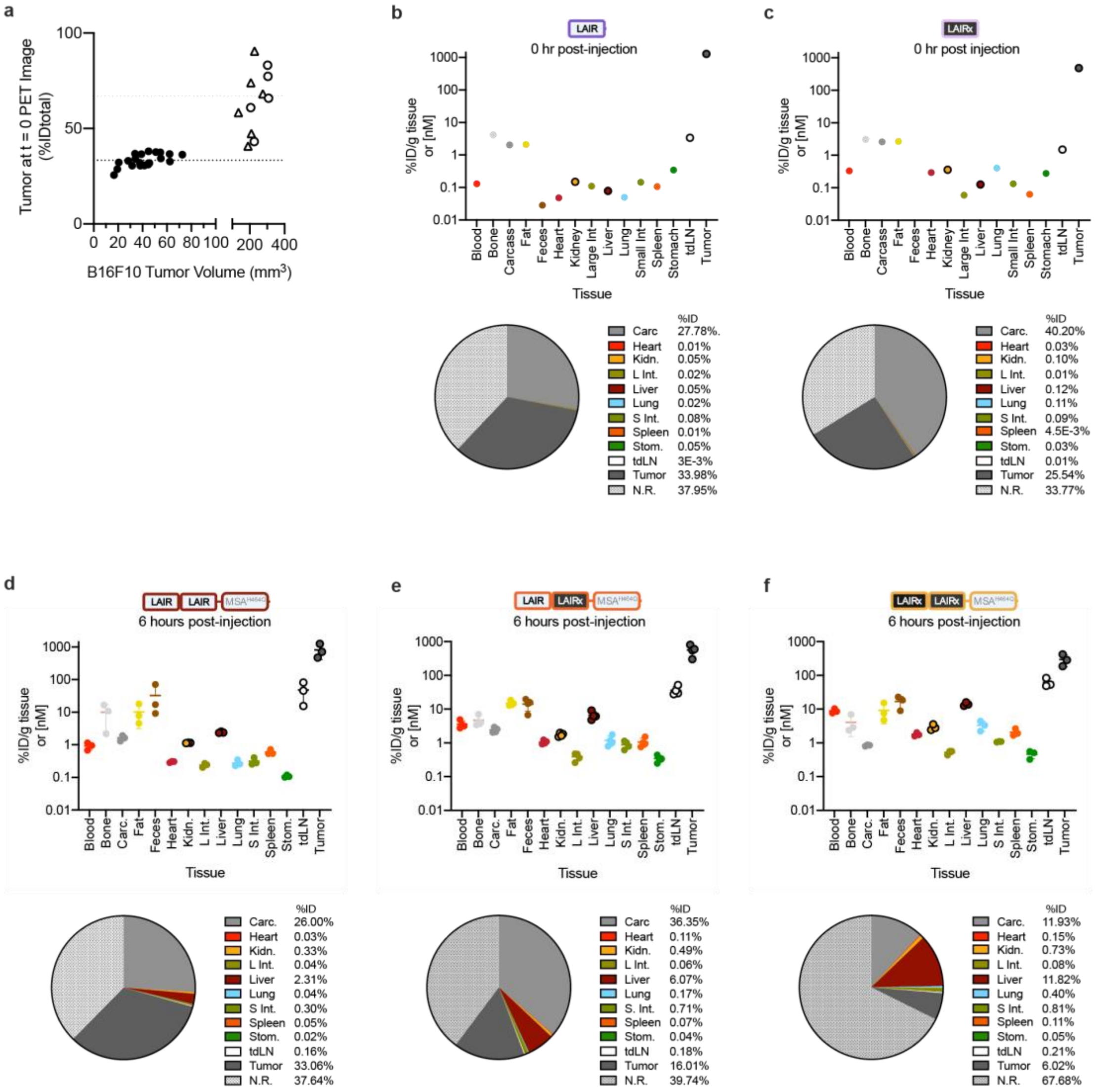

Supplementary Fig 14

Injected volume exceeding B16F10 hold-up volume leads to systemic drug dissemination.

a, Activity measured for small tumors in corrected PET images (filled circle, *n* = 21) taken immediately after 20 uL injection with radiolabeled proteins (in Supplementary Fig 12) or in large tumors homogenized immediately after 20 uL (open circles, *n* = 6) or 10 uL (open triangles, *n* = 9) injection with fluorescent mouse serum albumin. **b-c**, Activity measured in excised tissues by gamma-counter immediately after injection with b, LAIR (*n* = 1) and c, LAIRx (*n* = 1). **d-f**, Activity measured in excised tissues by gamma-counter 6 hours after injection with d, LAIR-LAIR-MSA^{H464Q} (mean +/- s.d., *n* = 3); **e**, LAIR-LAIR$_x$-MSA^{H464Q} (mean +/- s.d., *n* = 4); f, LAIR$_x$-LAIR$_x$-MSA^{H464Q} (mean +/- s.d., *n* = 3). Since 0.1 nmol of each protein was injected, the reported metric of %ID/g of tissue is numerically equivalent to the nanomolar concentration (nM) of protein in said tissue. ID, total injected dose; Carc., carcass devoid of excised organs; Kidn, kidneys; L. Int., large intestine; S. Int. small intestine; Stom., stomach; tdLN, tumor-draining lymph node; N.R., not recovered.

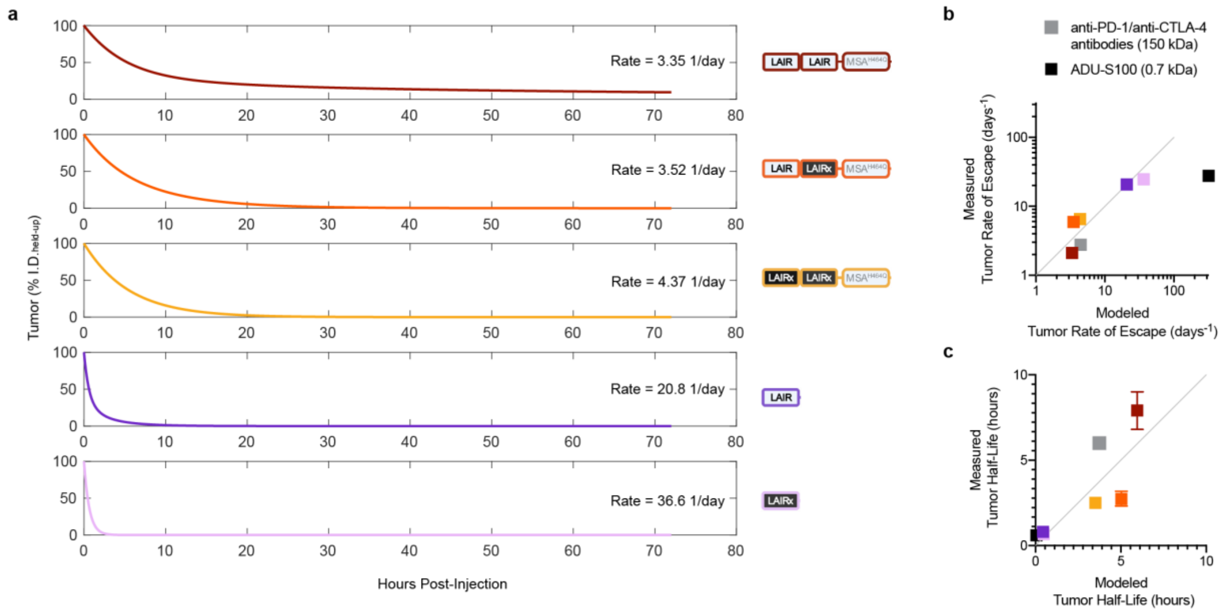

Supplementary Fig 15

Modeled rate of tumor escape of injected proteins agrees with PET measured rate of escape

Model simulation of **a,** the amount of fusion protein in the tumor over time after *in silico* intratumoral injection of LAIR-LAIR-MSA^{H464Q}, LAIR-LAIR$_x$-MSA^{H464Q}, LAIR$_x$-LAIR$_x$-MSA^{H464Q}, LAIR, and LAIR$_x$. Tumor rate of escape displayed on the graph is derived from curve fitting to a single-phase exponential decay. I.D.$_{\text{held-up}}$, injected dose held-up in the tumor immediately after intratumoral injection. **b-c**, Comparison of tumor b, rate of escape and c, residence half-life, which are related by the following expression [[half-life = ln(2)/(rate of escape)]), for each fusion protein predicted by the model, depicted in panel a; and measured *in vivo* (mean +/- s.d., *n* = 4 for all protein except *n* = 5 for LAIR-LAIR-MSA^{H464Q}), depicted in Fig 3c; and for anti-PD-1/anti-CTLA-4 antibodies reported by Francis et al. Sci Trans. Med., 2020[1] and ADU-S100 reported by Jang et al. Commun Biol. 2021[2] assuming a minimal collagen affinity.

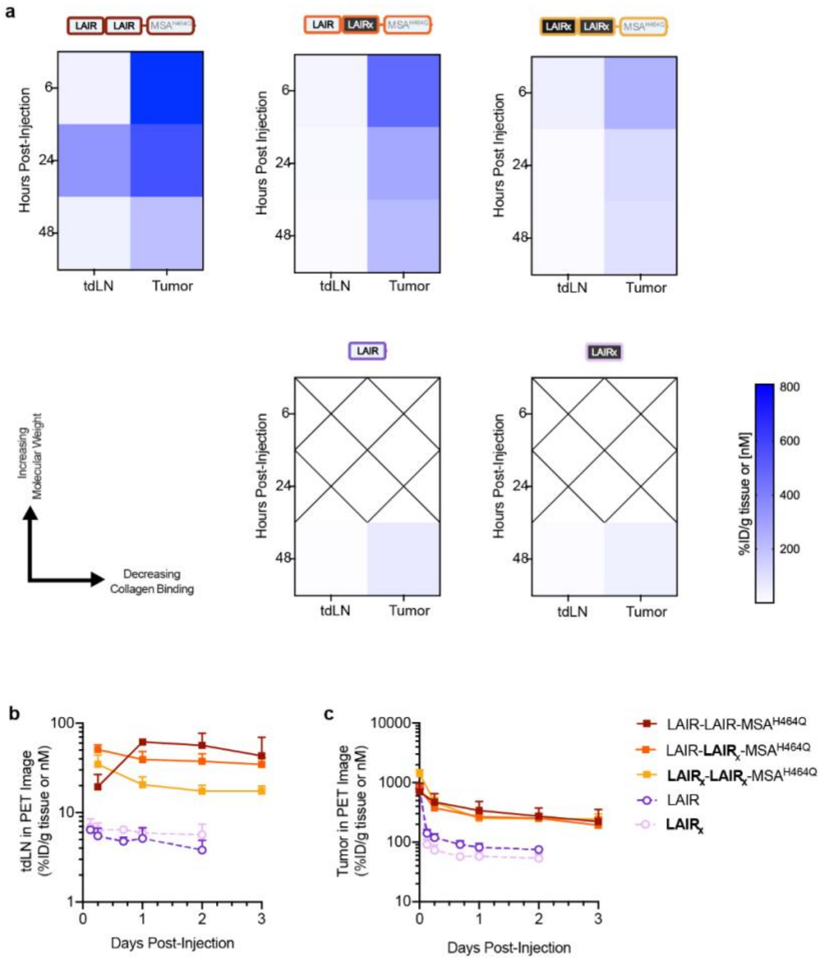

Supplementary Fig 16

Protein quantification from PET image validated by gamma counter measurement

a, Gamma counter quantification of ^{89}Zr-labeled fusion proteins LAIR-LAIR-MSA^{H464Q} (top left; *n* = 3 at 6 hour, *n* = 4 at 24 hours, *n* = 3 at 48 hours), LAIR-LAIR$_x$-MSA^{H464Q} (top middle; *n* = 4 at 6 hour, *n* = 4 at 24 hours, *n* = 4 at 48 hours), LAIR$_x$-LAIR$_x$-MSA^{H464Q} (top right; *n* = 3 at 6 hour, *n* = 3 at 24 hours, *n* = 3 at 48 hours), LAIR (bottom left; *n* = 4), LAIR$_x$ (bottom right; *n* = 4) remaining in tumor and tumor draining lymph node (tdLN) excised 6, 24 and 48 hours after intratumoral injection. ID, injected dose. Boxes containing X's designate data not collected. Quantification of designated ^{89}Zr-labeled fusion protein in the **b**, tdLN and **c**, tumor in isotope decay- and PVC-corrected PET images of B16F10 tumor-bearing mice at 0, 3, 6,17, 24, 48, 72 hours after intratumoral injection (mean + s.d., *n* = 5 for LAIR-LAIR-MSA^{H464Q} and LAIR-LAIR$_x$-MSA^{H464Q}, *n* = 4 for LAIR$_x$-LAIR$_x$-MSA^{H464Q}, LAIR, and LAIR$_x$)

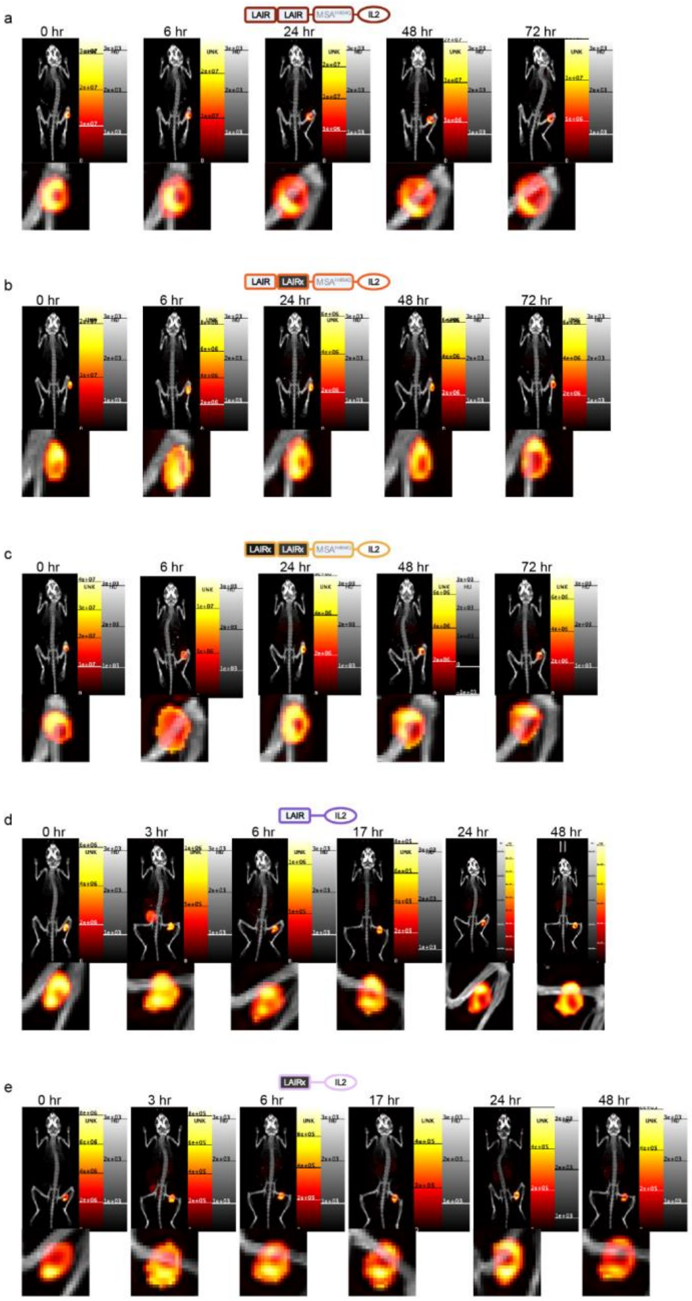

Supplementary Fig 17

Maximum intensity projection reveals peritumoral accumulation of intratumorally injected proteins

Representative isotope decay- and PVC-corrected whole body PET/CT 3D intensity projections of B16F10 tumor-bearing mice at 0, 3, 6, 17, 24, 48, and 72 hours after intratumoral injection of ^{89}Zr-labeled fusion proteins **a**, LAIR-LAIR-MSA^{H464Q} (n = 5), **b**, LAIR-LAIR$_x$-MSA^{H464Q} (n = 4), **c**, LAIR$_x$-LAIR$_x$-MSA^{H464Q} (n = 4 mice), **d**, LAIR (n = 4 mice), **e**, LAIR$_x$ (n = 4 mice). CT image (color bar in grayscale, Hounsfield units) is used for anatomical reference. PET emission (color bar in red-yellow standard uptake value [Bq/mL]) is different for each image, therefore not permissive for inter- and intra-group comparisons. Injected B16F10 tumors in whole body PET/CT 3D projections are enlarged to visualize injected proteins intratumoral distribution.

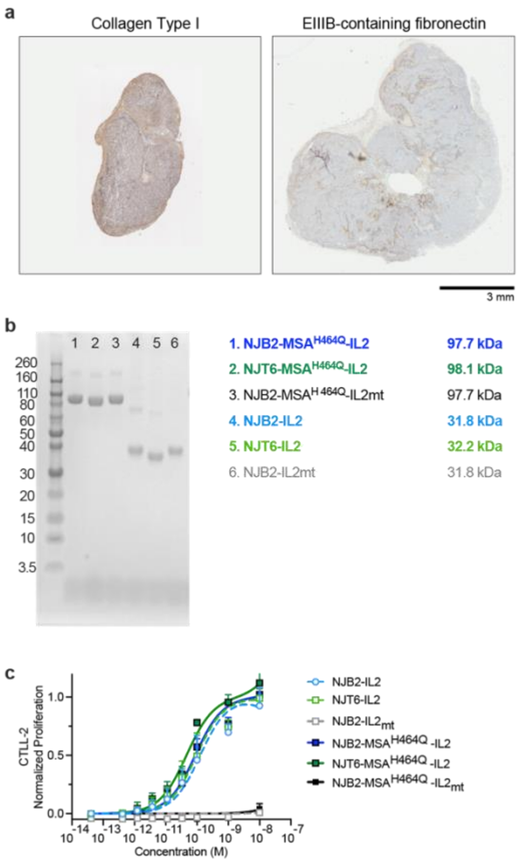

Supplementary Fig 18

Recombinant NJB2- and NJT6- IL-2 fusion proteins are monomeric and bioactive.

a, B16F10 tumors visualized for collagen type I (left) and EIIIB-containing fibronectin expression (right) by immunohistochemistry. Images are representative of three samples from two independent experiments. b, Purified IL-2 fusion proteins visualized by SDS-PAGE with Coomassie blue under non-reducing conditions. c, Dose-dependent CTLL-2 cell proliferation of IL-2 fusion proteins (mean + s.d.; $n = 3$). IL2mt denotes a mutant IL2 that renders the protein unable to bind to IL-2R; IL2mt containing proteins served as a negative control.

Supplementary Table 1: Glossary of compartment model variables and inputs

Symbol	Parameter	Value	Ref	Notes or Equation
**t**	Time	0 - 259200 s		This model simulates protein disposition for 72 hours after injection.
$\mathbf{V_{blood}}$	Blood Volume	~2 mL		Species and weight dependent. This model simulates protein disposition in a mouse.
$\mathbf{V_{tumor}}$	Tumor Volume	50 mm^3		This model simulates a B16F10 of 50 mm^3 in size.
$\mathbf{R_{cap}}$	Average tumor capillary radius	8 µm (5-15 µm)	3–5	In Krogh's cylinder model of vascular transport, $2R_{cap}/R_{Krogh}^2$ defines the blood vessel surface area (S) to tumor volume (V) ratio: $S/V = 2R_{cap}/R_{Krogh}^2$
$\mathbf{R_{Krogh}}$	Average radius of tissue surrounding tumor capillary	75 µm (20-150+ µm)	5	
**Molecular Size-Dependent Transport Parameters**				
$\mathbf{M_w}$	Molecular weight of agonist	Varies in model: 1 – 1000 kD		
$\mathbf{R_{mol}}$	Molecular radius of agonist	Varies in model: 0.3 - 100 nm	6	Dependent on the agonist's molecular size. Defined by this relationship: $\text{Log}(R_{mol}) = a + b \log(Mw)$, where R_{mol} is the molecular radius in nm; MW is the globular protein's molecular weight kD; and best fit yields, a = -0.31 and b = 0.43.
**ε**	Void fraction	Varies in model (0.05– 0.5)	7	Defined by the interstitial space accessible to an agonist, which is dependent on the agonist's molecular size and cellular packing of the tumor. This model implements a mathematical equation derived by Schmidt et al. relating void fraction (ε) and an agonist's molecular radius (R_{mol})
$\mathbf{k_{esc}}$	Rate of exchange between tumor and blood (balance of k_{intrav} and k_{extrav})	Varies	5,7	In Krogh's cylinder model of vascular transport, k_{esc} is defined by the permeability (P) surface area (S) product per tumor volume (V): $k_{esc} = PS/V = 2PR_{cap}/R_{Krogh}^2$
**P**	Tumor capillary permeability	Varies (1-150 $\times 10^{-9}$ m/s)	7	Dependent on an agonist's molecular size. This model uses a two-pore model of the vasculature in the tumor and implements a mathematical equation derived by Schmidt et al. that relates permeability (P) and molecular radius (R_{mol})
$\mathbf{k_{clear}}$	Rate of clearance from blood	Varies (0.1 – 5 hr^{-1})	7	Depends on an agonist's molecular size, globularity, charge, hydrophobicity, FcRn interactions, etc. This model implements a mathematical equation derived by Schmidt et al relating clearance rate (k_{clear}) and molecular radius (R_{mol}).

**LAIR and Collagen Binding Related Rates**				
$K_{d,collagen}$	Dissociation constant (affinity) of agonist and collagen	Varies (10^{-10} - 10^{-6} M)		$K_{d,col} = k_{off,col}/k_{on,col}$
$k_{on,col}$	Agonist's rate of association with collagen	10^{5} 1/M/s	8	Based on typical protein-protein interactions.
$k_{off,col}$	Agonist's rate of dissociation from collagen	Varies in model: 10^{-5} - 10^{-1} 1/s		$k_{off,col} = K_{d,col}k_{on,col}$
k_{deg}	Agonist's slow rate of degradation in tumor and blood	0.01 hr^{-1}		Based on measurement of proteins after incubation in 10% serum over time.
**IL-2 and IL-2R Binding Related Rates**				
$K_{d,IL\text{-}2R}$	Dissociation constant (affinity) of agonist and IL-2R	2.4×10^{-7} M	9	$K_{d,IL2} = k_{off,IL2R}/k_{on,IL2R}$
$k_{on,IL2R}$	Agonist'sIL-2 rate of association with IL-2RB	1.26×10^{6} 1/M/s	9	
$k_{off,IL2R}$	Agonist'sIL-2 rate of dissociation from IL-2RB	0.301 s^{-1}	9	
$k_{int,IL2R}$	Internalization rate of agonist-bound IL-2R	0.4 hr^{-1}	9	
$k_{prolif,IL2R}$	IL-2R+ cell proliferation rate	1 day^{-1} (0.1 - 3 day^{-1})	10	

Supplementary Table 2: Initial conditions inputted in compartment model

Definition	Value	Varies by	Estimate Derivation
**[C]₀**<br>Collagen in the **tumor**<br>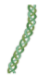	2×10^{-7} M	Tumor type	The acellular matrix contributes 44% of a B16F10 tumor mass and contains ~120 ng of hydroxyproline per mg. The remaining cellular fraction contains 9.5 ng of hydroxyproline per mg. On average, a B16F10 tumor contains ~58 ng of hydroxyproline per mg (0.44 x120 ng + 0.56 x 9.5 ng = 58 ng). Assuming a tissue density of 1 g/mL and hydroxyproline molar mass of 131 g/mol, a B16F10 tumor bears ~440 uM of hydroxyproline. Since a LAIR footprint spans 8-GPO triplets, three collagen fibrils comprise a collagen fiber, and nearly 100 fibers comprise a collagen bundle, the number of LAIR binding sites is approximately 2×10^{-7}M.<br>(440 uM/(8 x 3 x 100) ~ 0.2 uM)<br>(See supporting data in Supplementary Figure 11)
**$[L]_0$**<br>Injected LAIR-IL2 fusion protein in the **tumor**<br>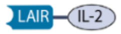	1×10^{-6} M	Injection volume & concentration. Tumor's size & void fraction.	The interstitial space of a 50 mm^3 B16F10 tumor with a void fraction of ~0.2 is 10 uL (50 mm^3 x 0.2 = 10 uL). Thus, initially only 10 uL of a 20 uL intratumoral injection would be retained intratumorally. For a 20 uL injection of 0.1 nmol in such tumor, the initial intratumoral concentration of injected protein is $[L]_{t=0} = 1 \times 10^{-6}$ M.<br>(0.1 nmol x 10 uL/20 uL)/50mm^3= 1×10^{-6} M)
**$[L_{blood}]_0$**<br>Injected LAIR-IL2 fusion protein in the blood<br>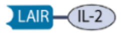	2.5×10^{-8} M	Injection volume & concentration. Tumor's size & void fraction.	Since 10 uL of 20 uL intratumoral injection comprising 0.1 nmol is instantaneously systemically distributed and the blood volume of a mouse is 2 mL, the blood concentration of injected LAIR-fusion protein is 2.5×10^{-8} M.<br>(0.1 nmol x 10 uL/20 uL)/2000 uL= 2.5×10^{-8} M)
**$[R]_0$**<br>IL-2R in the **tumor**<br>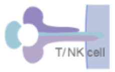	2.5×10^{-13} M	Varies by tumor's T and NK cell density	Six days after inoculation with 1 million cells, B16F10 tumors contain 150 x 10^3 T and NK cells per g tumor. Assuming an IL-2 receptor density of 1000 receptors/cell surface, B16F10 tumors contain ~1.5 x 10^8 IL-2R per g tumor. Assuming a tissue density of 1 g/mL, the IL-2R concentration is 2.5×10^{-13} M.<br>(1.5 x 10^8 IL-2R/g tumor x 1 g/mL)/(6.022E23) = 2.5×10^{-13} M)<br>(See supporting data in Supplementary Figure 6.)
**$[LC]_0$**<br>Injected protein-collagen complex in **tumor**<br>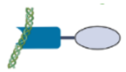	0 M		
**$[LR]_0$**<br>Injected protein-IL2R complex in the **tumor**<br>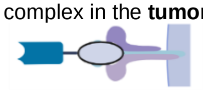	0 M		
**$[LCR]_0$**<br>Injected protein-collagen-IL2R complex in the **tumor**<br>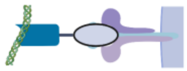	0 M		

Supplementary Table 3: Ordinary differential equations in the compartment model

Definition	Ordinary Differential Equation
**[C]**<br>Collagen in the **tumor**<br>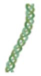	$\frac{d[C]}{dt} = \underbrace{k_{off,col}[LC] - k_{on,col}[C]\frac{[L]}{\varepsilon}}_{\text{Injected protein binding collagen}} + \underbrace{k_{off,col}[LCR] - k_{on,col}[C]\frac{[LR]}{\varepsilon}}_{\text{Injected protein-IL-2R complex binding collagen}} + \underbrace{k_{turn,col}[LC]}_{\text{Optional: TMDD collagen turnover}}$
**[L]**<br>Injected LAIR-IL2 fusion protein in the **tumor**<br>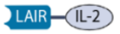	$\frac{d[L]}{dt} = \underbrace{k_{off,col}[LC] - k_{on,col}[C]\frac{[L]}{\varepsilon}}_{\text{Injected protein binding collagen}} + \underbrace{k_{off,IL2R}[LR] - k_{on,IL2R}[R]\frac{[L]}{\varepsilon}}_{\text{Injected protein binding IL-2R}} + \underbrace{k_{esc}\varepsilon([L_{blood}] - [L])}_{\text{Injected protein intravasating/extravasating}} - \underbrace{k_{deg}[L]}_{\text{Injected protein proteolysis}}$
**[L$_{blood}$]**<br>Injected LAIR-IL2 fusion protein in the blood<br>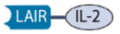	$\frac{d[L_{blood}]}{dt} = \underbrace{k_{esc}\varepsilon\left(\frac{[L]}{\varepsilon} - L_{blood}\right)\left(\frac{V_{tumor}\varepsilon}{V_{blood}}\right)}_{\text{Injected protein intravasating/extravasating}} - \underbrace{k_{clear}[L_{blood}]}_{\text{Injected protein cleared from blood}} - \underbrace{k_{deg}[L_{blood}]}_{\text{Injected protein proteolysis}}$
**[R]**<br>IL-2R in the **tumor**<br>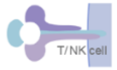	$\frac{d[R]}{dt} = \underbrace{k_{int,IL2R}[LR]}_{\text{Injected protein-IL-2R internalization}} + \underbrace{k_{prolif,IL2R}([R] + [LR] + [LCR])\left(1 - \frac{[R] + [LR] + [LCR]}{10^4[R]_0}\right)}_{\text{IL-2R cell expansion}} + \underbrace{k_{off,IL2R}[LR] - k_{on,IL2R}[R]\frac{[L]}{\varepsilon}}_{\text{Injected protein binding IL-2R}} + \underbrace{k_{off,IL2R}[LCR] - k_{on,IL2R}[R]\frac{[LC]}{\varepsilon}}_{\text{Injected protein-collagen complex binding IL-2R}}$
**[LC]**<br>Injected protein-collagen complex in **tumor**<br>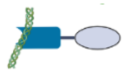	$\frac{d[LC]}{dt} = \underbrace{-k_{off,col}[LC] + k_{on,col}[C]\frac{[L]}{\varepsilon}}_{\text{Injected protein binding collagen}} + \underbrace{k_{off,IL2R}[LCR] - k_{on,IL2R}[R]\frac{[LC]}{\varepsilon}}_{\text{Injected protein-collagen complex binding IL-2R}} - \underbrace{k_{turn,col}[LC]}_{\text{Optional: TMDD collagen turnover}}$
**[LR]**<br>Injected protein-IL2R complex in the **tumor**<br>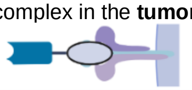	$\frac{d[LR]}{dt} = \underbrace{k_{off,col}[LCR] - k_{on,col}[C]\frac{[LR]}{\varepsilon}}_{\text{Injected protein-IL-2R complex binding collagen}} - \underbrace{k_{off,IL2R}[LR] + k_{on,IL2R}[R]\frac{[L]}{\varepsilon}}_{\text{Injected protein binding IL-2R}} - \underbrace{k_{int,IL2R}[LR]}_{\text{Injected protein-IL-2R internalization}}$
**[LCR]**<br>Injected protein-collagen-IL2R complex in the **tumor**<br>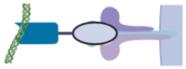	$\frac{d[LCR]}{dt} = \underbrace{-k_{off,col}[LCR] + k_{on,col}[C]\frac{[LR]}{\varepsilon}}_{\text{Injected protein-IL-2R complex binding collagen}} - \underbrace{k_{off,IL2R}[LCR] + k_{on,IL2R}[C]\frac{[LR]}{\varepsilon}}_{\text{Injected protein-IL-2R complex binding collagen}}$

Supplementary Table 4. Amino acid sequence of all novel fusion proteins

Fusion Protein Name	Amino Acid Sequence
LAIR	QEGSLPDITIFPNSSLMISQGTFVTVVCSYSDKHDLYNMVRLEKDGSTFMEKSTEPYKTEDE<br>FEIGPVNETITGHYSCIYSKGITWSERSKTLELKVIKENVIQTPAPGPTSDTSWLKTYSIYHHH<br>HHH
LAIR$_x$<br>(i.e.LAIR R41A E43A)	QEGSLPDITIFPNSSLMISQGTFVTVVCSYSDKHDLYNMVALAKDGSTFMEKSTEPYKTEDE<br>FEIGPVNETITGHYSCIYSKGITWSERSKTLELKVIKENVIQTPAPGPTSDTSWLKTYSIYHHH<br>HHH
LAIR-LAIR-MSA^{H464Q}	QEGSLPDITIFPNSSLMISQGTFVTVVCSYSDKHDLYNMVRLEKDGSTFMEKSTEPYKTEDE<br>FEIGPVNETITGHYSCIYSKGITWSERSKTLELKVIKENVIQTPAPGPTSDTSWLKTYSIYGG<br>GSQEGSLPDITIFPNSSLMISQGTFVTVVCSYSDKHDLYNMVRLEKDGSTFMEKSTEPYKT<br>EDEFEIGPVNETITGHYSCIYSKGITWSERSKTLELKVIKENVIQTPAPGPTSDTSWLKTYSIY<br>EAHKSEIAHRYNDLGEQHFKGLVLIAFSQYLQKCSYDEHAKLVQEVTDFAKTCVADESAAN<br>CDKSLHTLFGDKLCAIPNLRENYGELADCCTKQEPERNECFLQHKDDNPSLPPFERPEAEA<br>MCTSFKENPTTFMGHYLHEVARRHPYFYAPELLYYAEQYNEILTQCCAEADKESCLTPKLD<br>GVKEKALVSSVRQRMKCSSMQKFGERAFKAWAVARLSQTFPNADFAEITKLATDLTKVNK<br>ECCHGDLLECADDRAELAKYMCENQATISSKLQTCCDKPLLKKAHCLSEVEHDTMPADLP<br>AIAADFVEDQEVCKNYAEAKDVFLGTFLYEYSRRHPDYSVSLLLRLAKKYEATLEKCCAEA<br>NPPACYGTVLAEFQPLVEEPKNLVKTNCDLYEKLGEYGFQNAILVRYTQKAPQVSTPTLVE<br>AARNLGRVGTKCCTLPEDQRLPCVEDYLSAILNRVCLLQEKTPVSEHVTKCCSGSLVERRP<br>CFSALTVDETYVPKEFKAETFTFHSDICTLPEKEKQIKKQTALAELVKHKPKATAEQLKTVM<br>DDFAQFLDTCCKAADKDTCFSTEGPNLVTRCKDALAHHHHHH
LAIR-LAIR$_x$-MSA^{H464Q}	QEGSLPDITIFPNSSLMISQGTFVTVVCSYSDKHDLYNMVRLEKDGSTFMEKSTEPYKTEDE<br>FEIGPVNETITGHYSCIYSKGITWSERSKTLELKVIKENVIQTPAPGPTSDTSWLKTYSIYGG<br>GSQEGSLPDITIFPNSSLMISQGTFVTVVCSYSDKHDLYNMVALAKDGSTFMEKSTEPYKTE<br>DEFEIGPVNETITGHYSCIYSKGITWSERSKTLELKVIKENVIQTPAPGPTSDTSWLKTYSIYE<br>AHKSEIAHRYNDLGEQHFKGLVLIAFSQYLQKCSYDEHAKLVQEVTDFAKTCVADESAANC<br>DKSLHTLFGDKLCAIPNLRENYGELADCCTKQEPERNECFLQHKDDNPSLPPFERPEAEAM<br>CTSFKENPTTFMGHYLHEVARRHPYFYAPELLYYAEQYNEILTQCCAEADKESCLTPKLDG<br>VKEKALVSSVRQRMKCSSMQKFGERAFKAWAVARLSQTFPNADFAEITKLATDLTKVNKE<br>CCHGDLLECADDRAELAKYMCENQATISSKLQTCCDKPLLKKAHCLSEVEHDTMPADLPAI<br>AADFVEDQEVCKNYAEAKDVFLGTFLYEYSRRHPDYSVSLLLRLAKKYEATLEKCCAEANP<br>PACYGTVLAEFQPLVEEPKNLVKTNCDLYEKLGEYGFQNAILVRYTQKAPQVSTPTLVEAA<br>RNLGRVGTKCCTLPEDQRLPCVEDYLSAILNRVCLLQEKTPVSEHVTKCCSGSLVERRPCF<br>SALTVDETYVPKEFKAETFTFHSDICTLPEKEKQIKKQTALAELVKHKPKATAEQLKTVMDD<br>FAQFLDTCCKAADKDTCFSTEGPNLVTRCKDALAHHHHHH
LAIR$_x$-LAIR$_x$-MSA^{H464Q}	QEGSLPDITIFPNSSLMISQGTFVTVVCSYSDKHDLYNMVALAKDGSTFMEKSTEPYKTEDE<br>FEIGPVNETITGHYSCIYSKGITWSERSKTLELKVIKENVIQTPAPGPTSDTSWLKTYSIYGG<br>GSQEGSLPDITIFPNSSLMISQGTFVTVVCSYSDKHDLYNMVALAKDGSTFMEKSTEPYKTE<br>DEFEIGPVNETITGHYSCIYSKGITWSERSKTLELKVIKENVIQTPAPGPTSDTSWLKTYSIYE<br>AHKSEIAHRYNDLGEQHFKGLVLIAFSQYLQKCSYDEHAKLVQEVTDFAKTCVADESAANC<br>DKSLHTLFGDKLCAIPNLRENYGELADCCTKQEPERNECFLQHKDDNPSLPPFERPEAEAM<br>CTSFKENPTTFMGHYLHEVARRHPYFYAPELLYYAEQYNEILTQCCAEADKESCLTPKLDG<br>VKEKALVSSVRQRMKCSSMQKFGERAFKAWAVARLSQTFPNADFAEITKLATDLTKVNKE<br>CCHGDLLECADDRAELAKYMCENQATISSKLQTCCDKPLLKKAHCLSEVEHDTMPADLPAI<br>AADFVEDQEVCKNYAEAKDVFLGTFLYEYSRRHPDYSVSLLLRLAKKYEATLEKCCAEANP<br>PACYGTVLAEFQPLVEEPKNLVKTNCDLYEKLGEYGFQNAILVRYTQKAPQVSTPTLVEAA<br>RNLGRVGTKCCTLPEDQRLPCVEDYLSAILNRVCLLQEKTPVSEHVTKCCSGSLVERRPCF<br>SALTVDETYVPKEFKAETFTFHSDICTLPEKEKQIKKQTALAELVKHKPKATAEQLKTVMDD<br>FAQFLDTCCKAADKDTCFSTEGPNLVTRCKDALAHHHHHH
LAIR-LAIR-MSA^{H464Q}-IL2	QEGSLPDITIFPNSSLMISQGTFVTVVCSYSDKHDLYNMVRLEKDGSTFMEKSTEPYKTEDE<br>FEIGPVNETITGHYSCIYSKGITWSERSKTLELKVIKENVIQTPAPGPTSDTSWLKTYSIYGG<br>GSQEGSLPDITIFPNSSLMISQGTFVTVVCSYSDKHDLYNMVRLEKDGSTFMEKSTEPYKT<br>EDEFEIGPVNETITGHYSCIYSKGITWSERSKTLELKVIKENVIQTPAPGPTSDTSWLKTYSIY<br>EAHKSEIAHRYNDLGEQHFKGLVLIAFSQYLQKCSYDEHAKLVQEVTDFAKTCVADESAAN<br>CDKSLHTLFGDKLCAIPNLRENYGELADCCTKQEPERNECFLQHKDDNPSLPPFERPEAEA<br>MCTSFKENPTTFMGHYLHEVARRHPYFYAPELLYYAEQYNEILTQCCAEADKESCLTPKLD<br>GVKEKALVSSVRQRMKCSSMQKFGERAFKAWAVARLSQTFPNADFAEITKLATDLTKVNK<br>ECCHGDLLECADDRAELAKYMCENQATISSKLQTCCDKPLLKKAHCLSEVEHDTMPADLP<br>AIAADFVEDQEVCKNYAEAKDVFLGTFLYEYSRRHPDYSVSLLLRLAKKYEATLEKCCAEA<br>NPPACYGTVLAEFQPLVEEPKNLVKTNCDLYEKLGEYGFQNAILVRYTQKAPQVSTPTLVE

	AARNLGRVGTKCCTLPEDQRLPCVEDYLSAILNRVCLLQEKTPVSEHVTKCCSGSLVERRP<br>CFSALTVDETYVPKEFKAETFTFHSDICTLPEKEKQIKKQTALAELVKHKPKATAEQLKTVM<br>DDFAQFLDTCCKAADKDTCFSTEGPNLVTRCKDALAGGGSAPTSSSTSSSTAEAQQQQQ<br>QQQQQQQHLEQLLMDLQELLSRMENYRNLKLPRMLTFKFYLPKQATELKDLQCLEDELGP<br>LRHVLDLTQSKSFQLEDAENFISNIRVTVVKLKGSDNTFECQFDDESATVVDFLRRWIAFCQ<br>SIISTSPQHHHHHH
LAIR-LAIR$_x$-MSA^{H464Q}-IL2	QEGSLPDITIFPNSSLMISQGTFVTVVCSYSDKHDLYNMVRLEKDGSTFMEKSTEPYKTEDE<br>FEIGPVNETITGHYSCIYSKGITWSERSKTLELKVIKENVIQTPAPGPTSDTSWLKTYSIYGG<br>GSQEGSLPDITIFPNSSLMISQGTFVTVVCSYSDKHDLYNMVALAKDGSTFMEKSTEPYKTE<br>DEFEIGPVNETITGHYSCIYSKGITWSERSKTLELKVIKENVIQTPAPGPTSDTSWLKTYSIYE<br>AHKSEIAHRYNDLGEQHFKGLVLIAFSQYLQKCSYDEHAKLVQEVTDFAKTCVADESAANC<br>DKSLHTLFGDKLCAIPNLRENYGELADCCTKQEPERNECFLQHKDDNPSLPPFERPEAEAM<br>CTSFKENPTTFMGHYLHEVARRHPYFYAPELLYYAEQYNEILTQCCAEADKESCLTPKLDG<br>VKEKALVSSVRQRMKCSSMQKFGERAFKAWAVARLSQTFPNADFAEITKLATDLTKVNKE<br>CCHGDLLECADDRAELAKYMCENQATISSKLQTCCDKPLLKKAHCLSEVEHDTMPADLPAI<br>AADFVEDQEVCKNYAEAKDVFLGTFLYEYSRRHPDYSVSLLLRLAKKYEATLEKCCAEANP<br>PACYGTVLAEFQPLVEEPKNLVKTNCDLYEKLGEYGFQNAILVRYTQKAPQVSTPTLVEAA<br>RNLGRVGTKCCTLPEDQRLPCVEDYLSAILNRVCLLQEKTPVSEHVTKCCSGSLVERRPCF<br>SALTVDETYVPKEFKAETFTFHSDICTLPEKEKQIKKQTALAELVKHKPKATAEQLKTVMDD<br>FAQFLDTCCKAADKDTCFSTEGPNLVTRCKDALAGGGSAPTSSSTSSSTAEAQQQQQQQ<br>QQQQQHLEQLLMDLQELLSRMENYRNLKLPRMLTFKFYLPKQATELKDLQCLEDELGPLR<br>HVLDLTQSKSFQLEDAENFISNIRVTVVKLKGSDNTFECQFDDESATVVDFLRRWIAFCQSII<br>STSPQHHHHHH
LAIR$_x$-LAIR$_x$-MSA^{H464Q}-IL2	QEGSLPDITIFPNSSLMISQGTFVTVVCSYSDKHDLYNMVALAKDGSTFMEKSTEPYKTEDE<br>FEIGPVNETITGHYSCIYSKGITWSERSKTLELKVIKENVIQTPAPGPTSDTSWLKTYSIYGG<br>GSQEGSLPDITIFPNSSLMISQGTFVTVVCSYSDKHDLYNMVALAKDGSTFMEKSTEPYKTE<br>DEFEIGPVNETITGHYSCIYSKGITWSERSKTLELKVIKENVIQTPAPGPTSDTSWLKTYSIYE<br>AHKSEIAHRYNDLGEQHFKGLVLIAFSQYLQKCSYDEHAKLVQEVTDFAKTCVADESAANC<br>DKSLHTLFGDKLCAIPNLRENYGELADCCTKQEPERNECFLQHKDDNPSLPPFERPEAEAM<br>CTSFKENPTTFMGHYLHEVARRHPYFYAPELLYYAEQYNEILTQCCAEADKESCLTPKLDG<br>VKEKALVSSVRQRMKCSSMQKFGERAFKAWAVARLSQTFPNADFAEITKLATDLTKVNKE<br>CCHGDLLECADDRAELAKYMCENQATISSKLQTCCDKPLLKKAHCLSEVEHDTMPADLPAI<br>AADFVEDQEVCKNYAEAKDVFLGTFLYEYSRRHPDYSVSLLLRLAKKYEATLEKCCAEANP<br>PACYGTVLAEFQPLVEEPKNLVKTNCDLYEKLGEYGFQNAILVRYTQKAPQVSTPTLVEAA<br>RNLGRVGTKCCTLPEDQRLPCVEDYLSAILNRVCLLQEKTPVSEHVTKCCSGSLVERRPCF<br>SALTVDETYVPKEFKAETFTFHSDICTLPEKEKQIKKQTALAELVKHKPKATAEQLKTVMDD<br>FAQFLDTCCKAADKDTCFSTEGPNLVTRCKDALAGGGSAPTSSSTSSSTAEAQQQQQQQ<br>QQQQQHLEQLLMDLQELLSRMENYRNLKLPRMLTFKFYLPKQATELKDLQCLEDELGPLR<br>HVLDLTQSKSFQLEDAENFISNIRVTVVKLKGSDNTFECQFDDESATVVDFLRRWIAFCQSII<br>STSPQHHHHHH
LAIR-IL2	QEGSLPDITIFPNSSLMISQGTFVTVVCSYSDKHDLYNMVRLEKDGSTFMEKSTEPYKTEDE<br>FEIGPVNETITGHYSCIYSKGITWSERSKTLELKVIKENVIQTPAPGPTSDTSWLKTYSIYGG<br>GSAPTSSSTSSSTAEAQQQQQQQQQQQQHLEQLLMDLQELLSRMENYRNLKLPRMLTFK<br>FYLPKQATELKDLQCLEDELGPLRHVLDLTQSKSFQLEDAENFISNIRVTVVKLKGSDNTFE<br>CQFDDESATVVDFLRRWIAFCQSIISTSPQHHHHHH
LAIR$_x$-IL2	QEGSLPDITIFPNSSLMISQGTFVTVVCSYSDKHDLYNMVALAKDGSTFMEKSTEPYKTEDE<br>FEIGPVNETITGHYSCIYSKGITWSERSKTLELKVIKENVIQTPAPGPTSDTSWLKTYSIYGG<br>GSAPTSSSTSSSTAEAQQQQQQQQQQQQHLEQLLMDLQELLSRMENYRNLKLPRMLTFK<br>FYLPKQATELKDLQCLEDELGPLRHVLDLTQSKSFQLEDAENFISNIRVTVVKLKGSDNTFE<br>CQFDDESATVVDFLRRWIAFCQSIISTSPQHHHHHH
NJB2-IL2	QVQLVETGGGLVQAGGSLRLSCAASGSTFSHNAGGWYRQAPEKQRELVAGISSDGNINY<br>ADSVKDRFTISRDNASNTMYLQMNNLKPEDTAVYVCNIRGSYGNTYYSRWGQGTQVTVSS<br>GGGGGSAPTSSSTSSSTAEAQQQQQQQQQQQQHLEQLLMDLQELLSRMENYRNLKLPR<br>MLTFKFYLPKQATELKDLQCLEDELGPLRHVLDLTQSKSFQLEDAENFISNIRVTVVKLKGS<br>DNTFECQFDDESATVVDFLRRWIAFCQSIISTSPQLPETGGHHHHHH
NJB2-MSA^{H464Q}-IL2	QVQLVETGGGLVQAGGSLRLSCAASGSTFSHNAGGWYRQAPEKQRELVAGISSDGNINY<br>ADSVKDRFTISRDNASNTMYLQMNNLKPEDTAVYVCNIRGSYGNTYYSRWGQGTQVTVSS<br>GGEAHKSEIAHRYNDLGEQHFKGLVLIAFSQYLQKCSYDEHAKLVQEVTDFAKTCVADESA<br>ANCDKSLHTLFGDKLCAIPNLRENYGELADCCTKQEPERNECFLQHKDDNPSLPPFERPEA<br>EAMCTSFKENPTTFMGHYLHEVARRHPYFYAPELLYYAEQYNEILTQCCAEADKESCLTPK<br>LDGVKEKALVSSVRQRMKCSSMQKFGERAFKAWAVARLSQTFPNADFAEITKLATDLTKV

	NKECCHGDLLECADDRAELAKYMCENQATISSKLQTCCDKPLLKKAHCLSEVEHDTMPAD<br>LPAIAADFVEDQEVCKNYAEAKDVFLGTFLYEYSRRHPDYSVSLLLRLAKKYEATLEKCCAE<br>ANPPACYGTVLAEFQPLVEEPKNLVKTNCDLYEKLGEYGFQNAILVRYTQKAPQVSTPTLV<br>EAARNLGRVGTKCCTLPEDQRLPCVEDYLSAILNRVCLLQEKTPVSEHVTKCCSGSLVERR<br>PCFSALTVDETYVPKEFKAETFTFHSDICTLPEKEKQIKKQTALAELVKHKPKATAEQLKTVM<br>DDFAQFLDTCCKAADKDTCFSTEGPNLVTRCKDALAGGGSAPTSSSTSSSTAEAQQQQQ<br>QQQQQQQHLEQLLMDLQELLSRMENYRNLKLPRMLTFKFYLPKQATELKDLQCLEDELGP<br>LRHVLDLTQSKSFQLEDAENFISNIRVTVVKLKGSDNTFECQFDDESATVVDFLRRWIAFCQ<br>SIISTSPQLPETGGHHHHHH
NJT6-IL2	QVQLVETGGDLVQPGGSLRLSCAASGLTLDYYAIGWVRQAPGKEREGVSCITPQDGNTYY<br>DDSVMGRFTILRDNAKNMVYLQMNNLKPEDTAVYFCAAAGALTLDPSEYEYWGQGTQVT<br>VSSGGGGGSAPTSSSTSSSTAEAQQQQQQQQQQQQHLEQLLMDLQELLSRMENYRNLK<br>LPRMLTFKFYLPKQATELKDLQCLEDELGPLRHVLDLTQSKSFQLEDAENFISNIRVTVVKLK<br>GSDNTFECQFDDESATVVDFLRRWIAFCQSIISTSPQLPETGGHHHHHH
NJT6-MSA^{H464Q}-IL2	QVQLVETGGDLVQPGGSLRLSCAASGLTLDYYAIGWVRQAPGKEREGVSCITPQDGNTYY<br>DDSVMGRFTILRDNAKNMVYLQMNNLKPEDTAVYFCAAAGALTLDPSEYEYWGQGTQVT<br>VSSGGEAHKSEIAHRYNDLGEQHFKGLVLIAFSQYLQKCSYDEHAKLVQEVTDFAKTCVAD<br>ESAANCDKSLHTLFGDKLCAIPNLRENYGELADCCTKQEPERNECFLQHKDDNPSLPPFER<br>PEAEAMCTSFKENPTTFMGHYLHEVARRHPYFYAPELLYYAEQYNEILTQCCAEADKESCL<br>TPKLDGVKEKALVSSVRQRMKCSSMQKFGERAFKAWAVARLSQTFPNADFAEITKLATDL<br>TKVNKECCHGDLLECADDRAELAKYMCENQATISSKLQTCCDKPLLKKAHCLSEVEHDTM<br>PADLPAIAADFVEDQEVCKNYAEAKDVFLGTFLYEYSRRHPDYSVSLLLRLAKKYEATLEKC<br>CAEANPPACYGTVLAEFQPLVEEPKNLVKTNCDLYEKLGEYGFQNAILVRYTQKAPQVSTP<br>TLVEAARNLGRVGTKCCTLPEDQRLPCVEDYLSAILNRVCLLQEKTPVSEHVTKCCSGSLV<br>ERRPCFSALTVDETYVPKEFKAETFTFHSDICTLPEKEKQIKKQTALAELVKHKPKATAEQL<br>KTVMDDFAQFLDTCCKAADKDTCFSTEGPNLVTRCKDALAGGGSAPTSSSTSSSTAEAQQ<br>QQQQQQQQQQHLEQLLMDLQELLSRMENYRNLKLPRMLTFKFYLPKQATELKDLQCLED<br>ELGPLRHVLDLTQSKSFQLEDAENFISNIRVTVVKLKGSDNTFECQFDDESATVVDFLRRWI<br>AFCQSIISTSPQLPETGGHHHHHH

Supplementary References

1. Francis, D. M. *et al.* Blockade of immune checkpoints in lymph nodes through locoregional delivery augments cancer immunotherapy. *Sci. Transl. Med.* **12**, (2020).
2. Jang, S. C. *et al.* ExoSTING, an extracellular vesicle loaded with STING agonists, promotes tumor immune surveillance. *Commun. Biol.* **4**, 497 (2021).
3. Jain, R. K. Transport of molecules, particles, and cells in solid tumors. *Annu. Rev. Biomed. Eng.* **1**, 241–263 (1999).
4. Hilmas, D. E. & Gillette, E. L. Morphometric analyses of the microvasculature of tumors during growth and after x-irradiation. *Cancer* **33**, 103–110 (1974).
5. Wittrup, K. D., Thurber, G. M., Schmidt, M. M. & Rhoden, J. J. Practical theoretic guidance for the design of tumor-targeting agents. *Methods Enzymol.* **503**, 255–268 (2012).
6. Smilgies, D.-M. & Folta-Stogniew, E. Molecular weight–gyration radius relation of globular proteins: a comparison of light scattering, small-angle X-ray scattering and structure-based data. *J. Appl. Crystallogr.* **48**, 1604–1606 (2015).
7. Schmidt, M. M. & Wittrup, K. D. A modeling analysis of the effects of molecular size and binding affinity on tumor targeting. *Mol. Cancer Ther.* **8**, 2861–2871 (2009).
8. Tzeng, A., Kwan, B. H., Opel, C. F., Navaratna, T. & Wittrup, K. D. Antigen specificity can be irrelevant to immunocytokine efficacy and biodistribution. *Proc. Natl. Acad. Sci. U. S. A.* **112**, 3320–3325 (2015).
9. Charych, D. *et al.* Modeling the receptor pharmacology, pharmacokinetics, and pharmacodynamics of NKTR-214, a kinetically-controlled interleukin-2 (IL2) receptor agonist for cancer immunotherapy. *PLoS One* **12**, e0179431 (2017).

10. Kosmrlj, A. *et al.* Effects of thymic selection of the T-cell repertoire on HLA class I-associated control of HIV infection. *Nature* **465**, 350–354 (2010).
