# Supplementary material for: Maximizing response to intratumoral immunotherapy in mice by tuning local retention
Source: Nat Commun. 2022 Jan 10;13:109. doi: 10.1038/s41467-021-27390-6 (PMC8748612; doi:10.1038/s41467-021-27390-6)
Supplement: Supplementary file 2 — Reporting Summary [file 41467_2021_27390_MOESM2_ESM.pdf]

nature portfolio

Corresponding author(s): K. Dane Wittrup, Noor Momin

Last updated by author(s): Nov 11, 2021

Reporting Summary

Nature Portfolio wishes to improve the reproducibility of the work that we publish. This form provides structure for consistency and transparency in reporting. For further information on Nature Portfolio policies, see our Editorial Policies and the Editorial Policy Checklist.

Statistics

For all statistical analyses, confirm that the following items are present in the figure legend, table legend, main text, or Methods section.

n/a	Confirmed	
☐	☒	The exact sample size (n) for each experimental group/condition, given as a discrete number and unit of measurement
☐	☒	A statement on whether measurements were taken from distinct samples or whether the same sample was measured repeatedly
☐	☒	The statistical test(s) used AND whether they are one- or two-sided<br>*Only common tests should be described solely by name; describe more complex techniques in the Methods section.*
☒	☐	A description of all covariates tested
☐	☒	A description of any assumptions or corrections, such as tests of normality and adjustment for multiple comparisons
☐	☒	A full description of the statistical parameters including central tendency (e.g. means) or other basic estimates (e.g. regression coefficient) AND variation (e.g. standard deviation) or associated estimates of uncertainty (e.g. confidence intervals)
☐	☒	For null hypothesis testing, the test statistic (e.g. F, t, r) with confidence intervals, effect sizes, degrees of freedom and P value noted<br>*Give P values as exact values whenever suitable.*
☒	☐	For Bayesian analysis, information on the choice of priors and Markov chain Monte Carlo settings
☒	☐	For hierarchical and complex designs, identification of the appropriate level for tests and full reporting of outcomes
☒	☐	Estimates of effect sizes (e.g. Cohen's d, Pearson's r), indicating how they were calculated

Our web collection on statistics for biologists contains articles on many of the points above.

Software and code

Policy information about availability of computer code

Data collection	FACS data were obtained using the BD FACSDiva software (v7). ELISA plates and plate-based fluorescent/absorbance experiments were measured using Tecan Infinite M200 Pro absorbance/fluorescence plate reader and software. The PET images were acquired using a PerkimElmer (developed by Sofie) G8 PET/CT preclinical small-animal scanner. Tissue radioactivity was measured using a PerkinElmer 2480 Wizard2 automatic gamma counter. IHC stained tumor section were digitally scanned on a Leica Aperio AT2 Digital Pathology Slide Scanner (Leica Biosytems).
Data analysis	FlowJo 10.7.1 was used for analysis of FACS data. GraphPad Prism 9.0.2 was used for plotting and statistical analysis. MATLAB R2019b was used to conduct the modeling analysis. Partial volume correction (PVC) of the PET data was performed using PETPVC, an open source toolbox made available by the University College London (github.com/UCL). The PVC-corrected PET images were analyzed with AMIDE (v1.0.5) and VivoQuant software (v2.5; inviCRO, LLC, Boston, MA, USA) and Fiji (v2.1.0/1.53c). IHC stained tumor sections were analyzed on Aperio ImageScope (v12.3.0.5056). Word from Microsoft Office (v16.49) was used to draft the manuscript. Adobe Illustrator (v25.0.1) were used to assemble figures panels. The pharmacokinetic model, written and analyzed in MATLAB R2019b, has been published on Github (https://github.com/noormomin/Intratumoral-Immunotherapy-PK).

For manuscripts utilizing custom algorithms or software that are central to the research but not yet described in published literature, software must be made available to editors and reviewers. We strongly encourage code deposition in a community repository (e.g. GitHub). See the Nature Portfolio guidelines for submitting code & software for further information.

Data

Policy information about availability of data

All manuscripts must include a data availability statement. This statement should provide the following information, where applicable:

- Accession codes, unique identifiers, or web links for publicly available datasets
- A description of any restrictions on data availability
- For clinical datasets or third party data, please ensure that the statement adheres to our policy

The source data for all main and supplementary figures have been provided in a Source data file or on Github (https://github.com/noormomin/Intratumoral-Immunotherapy-PK). The crystal structure of murine LAIR-1 ectodomain was obtained from PDB 4ETY (accession code: http://doi.org/10.2210/pdb4ety/pdb). Any additional associated raw data or materials can be made available from the first author and corresponding author (N.M. and K.D.W.) on reasonable request. A reporting summary for this article is available as a Supplementary information file.

Field-specific reporting

Please select the one below that is the best fit for your research. If you are not sure, read the appropriate sections before making your selection.

- [x] Life sciences
- [ ] Behavioural & social sciences
- [ ] Ecological, evolutionary & environmental sciences

For a reference copy of the document with all sections, see nature.com/documents/nr-reporting-summary-flat.pdf

Life sciences study design

All studies must disclose on these points even when the disclosure is negative.

Sample size	Sample size was predetermined from past experiments to obtain statistically significant data (Momin et al., Sci. Transl. Med. 11, eaaw2614 (2019). In brief, sample sizes for tumor studies were calculated to detect at least a 30% difference between groups.
Data exclusions	No data were excluded from the study.
Replication	All in vitro experiments were performed at least twice for independent confirmation of results. Mouse tumor studies were done with the reported n, include pooled results from at least two independent experiments. PET/CT imaging was performed once for each time point with the indicated n. All other experiments were also done once for each time point with the indicated n. All attempts at replication were successful.
Randomization	Mice were randomized into treatment and imaging groups to provide the same mean tumor size at the start of treatment. Covariate control and correction is not relevant to this study because mice in this study were matched in age and tumor inoculation.
Blinding	No blinding was performed for the study. Collection of animal samples was not blinded because we needed to detail tumor area for each mouse in several different groups.

Reporting for specific materials, systems and methods

We require information from authors about some types of materials, experimental systems and methods used in many studies. Here, indicate whether each material, system or method listed is relevant to your study. If you are not sure if a list item applies to your research, read the appropriate section before selecting a response.

Materials & experimental systems

n/a	Involved in the study	
[ ]	[x]	Antibodies
[ ]	[x]	Eukaryotic cell lines
[x]	[ ]	Palaeontology and archaeology
[ ]	[x]	Animals and other organisms
[x]	[ ]	Human research participants
[x]	[ ]	Clinical data
[x]	[ ]	Dual use research of concern

Methods

n/a	Involved in the study	
[x]	[ ]	ChIP-seq
[ ]	[x]	Flow cytometry
[x]	[ ]	MRI-based neuroimaging

Antibodies

Antibodies used	Flow Cytometry on Tissue:<br>Rat IgG2b anti-mouse/human CD11b, BV 421, clone M1/70, Biolegend 101251<br>Rat IgG2c anti-mouse Ly6-C, PE-Cy7, clone HK1.4, Biolegend 128017<br>Rat IgG2a anti-mouse F4/80, PerCP-Cy5.5, clone BM8, Biolegend 123127

Rat IgG2b anti-mouse CD45, APC-Cy7, clone 30-F11, Biolegend 103115
Hamster anti-mouse CD3e, BUV 395, clone 145-2C11, BD Biosciences 563565
Rat anti-mouse CD8a, BUV 737, clone 53-6.7, BD Biosciences 612759
Mouse IgG2a anti-mouse NK1.1, PerCP-Cy5.5, clone PK136, Biolegend 108727
Rat IgG2b anti-mouse CD4, PE-Cy7, clone GK1.5, Biolegend 100421
Rat IgG1 anti-mouse CD25, Alexa Fluor 700, clone PC61, Biolegend 102024
Mouse IgG1 anti-mouse FoxP3, PE, clone 150D, Biolegend 320007
All antibodies were diluted 1:100 during staining.
Cell viability was assessed using Zombie Aqua Fixable Viability Kit (Biolegend 423101 at a 1:1000 dilution

Flow Cytometry and Sorting Yeast:
Polyclonal chicken anti- c-myc antibody, Gallus Immunotech ACMYC, at a 1:500 dilution
Streptavidin-Alexa Fluor 647, Invitrogen S21374, at a 1:200 dilution
Goat anti-chicken Alexa Fluor 488, Invitrogen A-11039, at a 1:200 dilution

Immunohistochemistry Staining:
Primary Staining for Collagen: Rabbit anti-collagen type I antibody, Abcam ab34710, , diluted 1:1000
Primary Staining for EIIIB-containing fibronectin: site-specifically biotinylated NJB2 (synthesized in-house)
Secondary Staining for Collagen: Goat anti-rabbit antibody HRP conjugated, Abcam ab6721, diluted at 1:1000
Secondary Staining for EIIIB-containing fibronectin: Streptavidin HRP-conjugated, Abcam ab64269, diluted at 1:1000

ELISA
anti-6xHis (ab1187, Abcam) at a 1:5000 dilution was used.
Treatments:
TA99 (synthesized in-house) was used at 100 ug per i.p. dose.

Validation

All purchase antibodies were validated by manufacturer (Biolegend, BD Biosciences, ThermoFisher, and Abcam) or published studies detailed on the following websites:
Rat IgG2b anti-mouse/human CD11b, BV 421, clone M1/70, Biolegend 101251: https://www.biolegend.com/en-us/products/brilliant-violet-421-anti-mouse-human-cd11b-antibody-7163?GroupID=BLG10427
Rat IgG2c anti-mouse Ly6-C, PE-Cy7, clone HK1.4, Biolegend 128017: https://www.biolegend.com/en-us/search-results/pe-cyanine7-anti-mouse-ly-6c-antibody-6063
Rat IgG2a anti-mouse F4/80, PerCP-Cy5.5, clone BM8, Biolegend 123127: https://www.biolegend.com/en-us/products/percp-cyanine5-5-anti-mouse-f480-antibody-4303?GroupID=BLG5319
Rat IgG2b anti-mouse CD45, APC-Cy7, clone 30-F11, Biolegend 103115: https://www.biolegend.com/en-us/products/apc-cyanine7-anti-mouse-cd45-antibody-2530?GroupID=BLG1932
Hamster anti-mouse CD3e, BUV 395, clone 145-2C11, BD Biosciences 563565: https://www.bdbiosciences.com/en-us/products/reagents/flow-cytometry-reagents/research-reagents/single-color-antibodies-ruo/buv395-hamster-anti-mouse-cd3e.563565
Rat anti-mouse CD8a, BUV 737, clone 53-6.7, BD Biosciences 612759: https://www.bdbiosciences.com/en-us/products/reagents/flow-cytometry-reagents/research-reagents/single-color-antibodies-ruo/buv737-rat-anti-mouse-cd8a.612759
Mouse IgG2a anti-mouse NK1.1, PerCP-Cy5.5, clone PK136, Biolegend 108727: https://www.biolegend.com/en-us/search-results/percp-cyanine5-5-anti-mouse-nk-11-antibody-4289?GroupID=GROUP20
Rat IgG2b anti-mouse CD4, PE-Cy7, clone GK1.5, Biolegend 100421: https://www.biolegend.com/en-us/products/pe-cyanine7-anti-mouse-cd4-antibody-1919?GroupID=BLG4211
Rat IgG1 anti-mouse CD25, Alexa Fluor 700, clone PC61, Biolegend 102024: https://www.biolegend.com/en-us/products/alexa-fluor-700-anti-mouse-cd25-antibody-3389?GroupID=BLG10428
Mouse IgG1 anti-mouse FoxP3, PE, clone 150D, Biolegend 320007: https://www.biolegend.com/en-us/products/pe-anti-mouse-rat-human-foxp3-antibody-2889?GroupID=BLG4178
Zombie Aqua Fixable Viability Kit Biolegend 423101 at a 1:1000 dilution: https://www.biolegend.com/en-us/products/zombie-aqua-fixable-viability-kit-8444?GroupID=BLG2181

Flow Cytometry and Sorting Yeast:
Chicken anti- c-myc antibody, Gallus Immunotech ACMYC, at a 1:500 dilution: https://www.thermofisher.com/antibody/product/C-MYC-Epitope-Tag-Antibody-Polyclonal/ACMYC
Streptavidin-Alexa Fluor 647, Invitrogen S21374, at a 1:200 dilution: https://www.thermofisher.com/order/catalog/product/S21374#/S21374
Goat anti-chicken Alexa Fluor 488, Invitrogen A-11039, at a 1:200 dilution: https://www.thermofisher.com/antibody/product/Goat-anti-Chicken-IgY-H-L-Secondary-Antibody-Polyclonal/A-11039

Immunohistochemistry Staining:
Rabbit anti-collagen type I antibody, Abcam ab34710, , diluted 1:1000: https://www.abcam.com/collagen-i-antibody-ab34710.html
Goat anti-rabbit antibody HRP conjugated, Abcam ab6721, diluted at 1:1000: https://www.abcam.com/goat-rabbit-igg-hl-hrp-ab6721.html
Streptavidin HRP-conjugated, Abcam ab64269, diluted at 1:1000: https://www.abcam.com/streptavidin-hrp-ready-to-use-ab64269.html

TA99 was synthesized in-house and validated by a flow cytometry B16F10 binding assay and several previous publications (Zhu, Gai, Opel et al. Cancer Cell. 13, 27(4):489-501. (2015), Momin et al., Sci. Transl. Med. 11, eaaw2614 (2019), etc.)

NJB2 specificity was validated in a previous publication Jailkhani et al., Proc. Natl. Acad. Sci. U.S.A.116(28)14181-14190. (2019).

Eukaryotic cell lines

Policy information about cell lines

Cell line source(s)	Cell lines B16F10 (ATCC), HEK293-F (Gibco), and CTLL-2 (ATCC) cells were cultured following vendor instructions.
Authentication	The cell lines arrived from Gibco and ATCC with authentication and characterization and have been validated by several publications:<br>B16F10 (ATCC): https://www.atcc.org/products/crl-6475<br>HEK293-F (Gibco): https://www.thermofisher.com/order/catalog/product/R79007#/R79007<br>CTLL-2 (ATCC): https://www.atcc.org/products/tib-214<br>Each cell line was maintained separately and stocked in early passages to minimize contamination and keep identity.
Mycoplasma contamination	All cell-lines that were inoculated in mice were confirmed without mycoplasma contamination by PCR.
Commonly misidentified lines (See ICLAC register)	No commonly misidentified cell lines were used.

Animals and other organisms

Policy information about studies involving animals; ARRIVE guidelines recommended for reporting animal research

Laboratory animals	Female C57BL/6 (Taconic, C57BL/6NTac) at 6-10 weeks age were purchased and maintained in the animal facility at the Massachusetts Institute of Technology. Mice were fed normal chow and water ad libitum in a pathogen-free vivarium under standard conditions (temperature around 22 C; relative humidity of 40-70% and a 12-hour light-dark cycle)
Wild animals	No wild animals were used in this study
Field-collected samples	This study did not involve samples collected from the field
Ethics oversight	All animal studies and procedures were carried out following federal, state and local guidelines under an Institutional Animal Care and Use Committee (IACUC) approved animal protocol by the MIT Committee of Animal Care (CAC) and Division of Comparative Medicine.

Note that full information on the approval of the study protocol must also be provided in the manuscript.

Flow Cytometry

Plots

Confirm that:

- [x] The axis labels state the marker and fluorochrome used (e.g. CD4-FITC).
- [x] The axis scales are clearly visible. Include numbers along axes only for bottom left plot of group (a 'group' is an analysis of identical markers).
- [x] All plots are contour plots with outliers or pseudocolor plots.
- [x] A numerical value for number of cells or percentage (with statistics) is provided.

Methodology

Sample preparation	B16F10 tumors harvested after i.t. treatment were mechanically digested through 70 um nylon cell strainers to prepare single-cell suspensions. Yeast cells were filtered through a 70 um nylon cell strainer to prepare single-cell suspension for sorting and analysis. All single-cell suspension tissue samples and yeast were then resuspended in ice-cold PBS containing 0.1% (w/v) BSA and normalized to the weight of tissue per sample or optimal density, respectively, before staining.
Instrument	Cells were analyzed using BD FACS LSR Fortessa, or sorted on the BD FACSAria III Cell Sorter.
Software	BD FACSDiva (BD Biosciences) was used for collection of FACS data and FlowJo was used for analysis. The collected data was plotted with statistical analysis by GraphPad Prism.
Cell population abundance	Enrichment of clones using yeast display requires sorting cells representing 0.1 - 1% of the total population.
Gating strategy	The general gating strategy used for all relevant experiments entails a preliminary FSC/SCC gate to differentiate cells from debris followed by a FSC-A/FSC-H and SSC-A/SSC-H gated to differentiate single cells from doublets. A physical parameter and the fixable viability stain Zombie Aqua were used to exclude dead cells from analysis. Positive populations were defined using not stained cells (FMO samples) as reference.<br>For Figure 1, the yeast sort gating is provided in panel b.<br>For Extended Data Figure 10, the representative gating is provided in panel c.

- [x] Tick this box to confirm that a figure exemplifying the gating strategy is provided in the Supplementary Information.
